# Joint estimation of preferential attachment and node fitness in growing complex networks

**DOI:** 10.1038/srep32558

**Published:** 2016-09-07

**Authors:** Thong Pham, Paul Sheridan, Hidetoshi Shimodaira

**Affiliations:** 1 Division of Mathematical Science, Graduate School of Engineering Science, Osaka University, Osaka, Japan; 2 The Institute of Medical Science, The University of Tokyo, Tokyo, Japan

## Abstract

Complex network growth across diverse fields of science is hypothesized to be driven in the main by a combination of preferential attachment and node fitness processes. For measuring the respective influences of these processes, previous approaches make strong and untested assumptions on the functional forms of either the preferential attachment function or fitness function or both. We introduce a Bayesian statistical method called PAFit to estimate preferential attachment and node fitness without imposing such functional constraints that works by maximizing a log-likelihood function with suitably added regularization terms. We use PAFit to investigate the interplay between preferential attachment and node fitness processes in a Facebook wall-post network. While we uncover evidence for both preferential attachment and node fitness, thus validating the hypothesis that these processes together drive complex network evolution, we also find that node fitness plays the bigger role in determining the degree of a node. This is the first validation of its kind on real-world network data. But surprisingly the rate of preferential attachment is found to deviate from the conventional log-linear form when node fitness is taken into account. The proposed method is implemented in the R package PAFit.

The study of complex network evolution is a hallmark of network science. Research in this discipline is inspired by empirical observations underscoring the widespread nature of certain structural features, such as the small-world property^1^, a high clustering coefficient^2^, a heavy tail in the degree distribution^3^, assortative mixing patterns among nodes^4^, and community structure^5^ in a multitude of biological, societal, and technological networks^6^,^7^,^8^,^9^,^10^,^11^. Network scientists actively seek to explain these sorts of structural features held in common among complex networks across diverse domains of learning in terms of the ordinary operation of simple mechanistic processes.

An extensive body of literature on the mechanisms of complex network evolution has been amassed in the time since the subject first began to flourish around the turn of the century^12^,^13^,^14^. Various mechanisms have been advanced, including preferential attachment^15^, node fitness^16^, node duplication combined with edge duplication and divergence^17^, homophily^18^, topological distance^19^, and node birth/death processes^20^. Among them, preferential attachment and node fitness have garnered special attention, not only because they are the first mechanisms that were proposed to explain structural features observed in real-world complex networks, but also for their easy and attractive interpretations. *Preferential attachment* (PA) is a “rich-get-richer” mechanism^21^,^22^ according to which the amount of some quantity distributed among the members of a population increases with the amount of the quantity they already possess. This is in contrast to *fitness*, which is a “fit-get-richer” effect, whereby the ability of individuals in a population to acquire a given quantity is determined by intrinsic qualities. In this process, the larger the fitness an individual has, the more likely it will be that the individual prospers. Individual node fitness may differ, and thus represent heterogeneity in a population.

Network scientists rely on a class of network models, known as *generative network models*, or sometimes evolving or growing network models, to investigate possible mechanisms underlying complex network formation. In this modelling paradigm, complex networks are generated by means of the incremental addition and deletion of nodes and edges to a seed network over a long sequence of time-steps. This sequence is denoted by 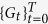 with *G*_0_ the seed, and *G*_*T*_ the final network. Figure 1 shows an example of a growing network, which is a special kind of generative network model that is defined by a sequence of additions of nodes and edges. The mechanisms according to which a complex network evolves are captured by transition rules governing how *G*_*t*−1_ transits to *G*_*t*_ at time-step *t* for *t* ≥ 1. The rationale behind the study of these models is that if the mechanisms governing node/edge dynamics in a given model produce networks with structural features similar on average to those observed in real networks, then it is within the bounds of possibility that the same mechanisms are also operative in their real-world counterparts.

The Barabási-Albert (BA) model^15^, which is closely related to the older Price’s model^23^, is the most widely known PA based growing network model. It is defined by a simple form of PA in which the probability that a node *v*_*i*_ of degree *k*_*i*_(*t*) = *k* acquires an edge at time-step *t* is defined to be proportional to *A*_*k*_ = *k*. The time-independent function *A*_*k*_ is known as the *PA function*. Historically, the term PA was often used to refer to this special case. But any *A*_*k*_ that increases with *k* on average is in keeping with the spirit of “preferential attachment”. Thus in this paper, we will use the term rich-get-richer and PA interchangeably to describe the situation when *A*_*k*_ is a function that increases with *k* on average. The functional form of *A*_*k*_ has been shown to affect network structure, in particular degree distribution. In a generalisation of the BA model where *A*_*k*_ takes the popular log-linear form *k*^*α*^ for *attachment exponent α* > 0, it has been shown that each of the linear (*α* = 1), sub-linear (*α* < 1) and super-linear (*α* > 1) sub-cases result in networks with different asymptotic degree distributions^11^,^15^,^24^. In particular, the case *α* = 1 generates *scale-free* networks, which is a class of networks whose frequency of a node with degree *k* takes the power-law functional form *k*^−*γ*^ with some positive scaling exponent *γ*. Although there are some arguments whether real-world networks really are scale-free^3^,^25^,^26^,^27^, the scale-free property nevertheless serves as an important and founding notion when discussing structural properties of complex networks.

Generative network models based on the fitness mechanism have also been shown to give rise to scale-free networks^16^,^28^,^29^,^30^. The model of Caldarelli *et al*.^28^ is the most basic model of this kind. In mathematical terms, each node *v*_*i*_ acquires new connections with probability proportional to *η*_*i*_. The time-independent fitness *η*_*i*_ is conventionally interpreted as the intrinsic excellence of node *v*_*i*_. It is important to note that *η*_*i*_ is assumed to be independent of any graph theoretic properties, such as node degree. In this paper, we will use the terms fit-get-richer and fitness mechanism interchangeably.

Attempts have been made to unify PA and node fitness in a single model. Bianconi and Barabási (BB)^16^ model both PA and node fitness, however, the definition of PA is restricted to that of the original BA model. The General Temporal (GT) model^31^ stochastically models both rich-get-richer and fit-get-richer processes by defining the probability that a node *v*_*i*_ with degree *k*_*i*_(*t*) = *k* receives new links at time-step *t* to be proportional to the product:


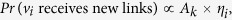


where *A*_*k*_ is a function of degree *k* and *η*_*i*_ the fitness of node *v*_*i*_, respectively. Note that while *A*_*k*_ and *η*_*i*_ are assumed to be time-invariant, that is, *A*_*k*_(*t*) = *A*_*k*_ and *η*_*i*_(*t*) = *η*_*i*_ for every degree *k*, node *i* and time-step *t*, the number of new edges *m*(*t*) and number of new nodes *n*(*t*) at each time-step are free to vary. The GT model includes all of the models mentioned above and more as special cases^15^,^16^,^24^,^28^,^32^,^33^. The landscape of these models is surveyed in Table 1. Holme^34^ provides a recent review of some other temporal network models.

It is generally assumed that a mixture of PA and fitness drive complex network evolution^16^,^35^,^36^,^37^. But any such mechanism, or combination thereof, no matter how plausible, must be empirically validated using specially designed statistical techniques in order to meet the burdens of science. However, the current crop of statistical estimation methods assumes one of these special cases of the GT model, but never the full model itself. As a result they either ignore the effect of PA or node fitness completely^19^,^31^,^38^,^39^,^40^,^41^,^42^,^43^,^44^,^45^, or otherwise assume the existence of one in a highly constrained form, and work to estimate the other^29^,^35^,^46^. For the problem of estimating fitness in the time-invariant case, which is the closest to our setting here, Kong *et al*.’s growth method^29^ is the only existing method we know that estimates *η*_*i*_, albeit under the assumption that *A*_*k*_ = *k*. More details on related works are provided in the Supplementary Information Section S2.1.

The questions as to how PA and node fitness mechanisms could be validated and quantified boil down to the following statistical estimation problem: how are the PA function *A*_*k*_ and node fitnesses *η*_*i*_ to be estimated from observed network data? It is important to note that no existing work considers the detection or estimation of the joint presence of these rich-get-richer and fit-get-richer effects.

Contrary to previous work, by assuming the GT model in its general form, we let the data speak for itself as regards the quantification of both rich-get-richer and fit-get-richer effects without imposing any assumptions on the functional forms of *A*_*k*_ and fitness distribution *P*(*η*). For example, we address such questions as: is there evidence for PA in real-world networks even after having taken node fitness into account, and vice versa? Another motivation for estimating these effects is that even a rough understanding of the functional forms of *A*_*k*_ and *P*(*η*) is liable to provide valuable insights into the global characteristics of complex networks. An important theoretical question then arises as to whether the widely accepted log-linear form in *k* is true of real-world networks, or does *A*_*k*_ take other more exotic forms?

Analogous questions arise in the context of fitness. When *A*_*k*_ is linear, it has been shown that bounded distributions of node fitness give rise to a power-law degree distribution with different scaling exponents, while unbounded distributions lead to a “winner-takes-all” scenario, in which a single node absorbs all the newly incoming edges^16^,^29^,^30^. So it is only natural to ask what kind of empirical distributions of node fitness exist in real-world complex networks, after we have allowed the simultaneous estimation of *A*_*k*_ free of any assumption on its functional form?

Last but not least, the jointly estimated *A*_*k*_ and *η*_*i*_ may more accurately reflect the evolutionary mechanisms of a network, than those obtained from a method that estimates either *A*_*k*_ and *η*_*i*_ in isolation, and can be exploited in practical problems. For example, using the estimation result, we are able to calculate the probability a given node receives new links in link prediction problems^49^,^50^. Moreover, the *η*_*i*_’s are of particular interests in their own right. Using the *η*_*i*_’s, it is possible to identify the nodes that are really “attractive” based on their intrinsic excellence, after having accounted for the rich-get-richer effect described by the *A*_*k*_ function. This might be of considerably interest, for example, in identifying research papers that have real value^35^,^37^.

Our main contributions are two-fold. The first contribution is a statistical method called PAFit to simultaneously estimate the PA and node fitness functions without imposing any assumptions on their functional forms. To the best of our knowledge, PAFit is the first ever method in the literature that can do so. Even though there are recent works^35^,^44^,^45^,^46^ that employ a time-varying PA function or node fitness, which at first glance appears to be more general than our time-invariant setting, all of these works assumed the presence of PA and fitness with functional forms imposed *a priori*, and thus cannot answer the very question about the co-existence of PA and fitness, as well as their true functional forms. While our time-invariant setting may seem to be restrictive, the nonparametric nature of our method makes it an important first step towards a truly nonparametric time-varying method, if such a method is possible.

In PAFit, we take a Bayesian approach, and formulate the estimation problem as the maximization of the log-likelihood function of the GT model with suitably added regularization terms to avoid overfitting. The regularization terms can be interpreted as Bayesian prior distributions of the parameters. Thus the estimated (*A*_*k*_, *η*_*i*_) is the Maximum-a-Posteriori (MAP) estimate from Bayesian inference. For statistically reliable results, we also implement logarithmic binning over the degrees when estimating the PA function^31^. We then provide a Minorize-Maximization (MM) algorithm^51^ to efficiently solve the maximization problem. Using the inverse of the negative Hessian matrix of the log posterior calculated at the MAP, our method can also provide approximate credible intervals for the estimated values. The proposed method is implemented in the R package PAFit^52^. For a tutorial of how to use the package, see the accompanying vignette^53^.

Our PAFit method contains two regularization parameters: *r* (PA regularization parameter) and *s* (fitness regularization parameter). The parameter *r* controls the amount of regularization for the PA function in so far as the bigger the value of *r*, the more *A*_*k*_ assumes the form *k*^*α*^. On the other hand, 1/*s* is the variance of a gamma prior distribution over *P*(*η*) with mean 1. As will be shown in the Methods Section, each scenario of the co-existence of PA and fitness (e.g. PA only, fitness only, or both PA and fitness, and their assumed functional forms) corresponds to a particular combination of the regularization parameters *r* and *s* (see Table 2).

In order to choose the optimal *r* and *s* for a particular dataset, we use the common approach of splitting the dataset into two parts: a learning part and a testing part. Recall that the full dataset consists of time-steps collected sequentially. In this paper, we set the value of *p*, that is, the ratio of the number of new edges in the learning data and the full data, to 0.75. This can be done by taking the first three-quarters of the full dataset (in terms of number of new edges) as the learning data, and taking the remaining last quarter be the testing data. We estimate *A*_*k*_ and *η*_*i*_ of the GT model for every combination of *r* and *s* on some grid *D* using the learning data, and then measure the likelihood of the testing data. It is important to note that the testing part is unseen in the learning phase. Thus a model with a large number of parameters does not necessarily give higher likelihood in the testing part than a model with smaller number of parameters. The workflow of the PAFit method is summarized in Fig. 2. More details are provided in the Methods Section.

In our second contribution, we report the first evidence of the co-existence of PA and fitness mechanisms, or, in other words, rich-get-richer and fit-get-richer effects in the growth of a Facebook wall-post dataset^54^. While this confirms our expectation that there can be a mixture of two effects driving complex network evolution, we go further and show that, in this dataset, the fit-get-richer is actually the stronger of the two effects in governing the degree of a node. We also show that, contrary to the popular assumption of a log-linear PA function, the estimated *A*_*k*_ turned out to be highly non-log-linear. These estimated *A*_*k*_ become flat in the high-degree region. This might indicate a limit in our capacity to make new acquaintances or new collaborations^55^. Given that most existing works have modeled the PA function as log-linear in *k* at best, and a substantial body of previous works even assume *A*_*k*_ to be linear, this important finding calls for a need to consider more general functional forms.

## Results

### An illustrative example

Here we present two simulated examples to demonstrate the workings of our proposed methodology. In the first example, the true PA function is *A*_*k*_ = max(*k*, 1), which is the widely-popular linear PA function. The second example uses the true PA function *A*_*k*_ = 3(log max(*k*, 1))^2^ + 1, which presents a non-log-linear function that deviates from conventional assumptions. Other examples with different functional forms are considered in the Supplementary Information Section S1.1. Note that these are true functions used for simulation, not that our PAFit method needs to use any information about them in the estimation. Starting from a seed network with 100 nodes, *m*(*t*) = 5 new edges and *n*(*t*) = 1 new node are added at each time-step *t* until the total number of nodes reached is *N* = 10000. The true underlying node fitnesses are sampled from a gamma distribution with mean 1 and variance 1/s*. Here we set s* = 1. We note that in this case the distribution is also an exponential distribution with mean 1.

We compare PAFit with the growth method of Kong *et al*.^29^, which is designed to estimate node fitness, albeit under the assumption that *A*_*k*_ is equal to *k*. The growth method is the closest existing work to our setting. We use the following three metrics to measure how well the methods perform: the average relative error in estimating node fitness, defined as 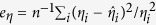 where *n* is the number of nodes that we estimated fitness for; the average relative error in estimating the PA function, defined as 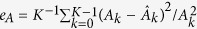 where *K* is the maximum degree that appears in the growth process of the network; and, finally, the correlation *r*_*η*_ between true and estimated fitness. In both methods we only estimate fitness of nodes that acquired at least five new edges in the growth process.

**Figure 2:**
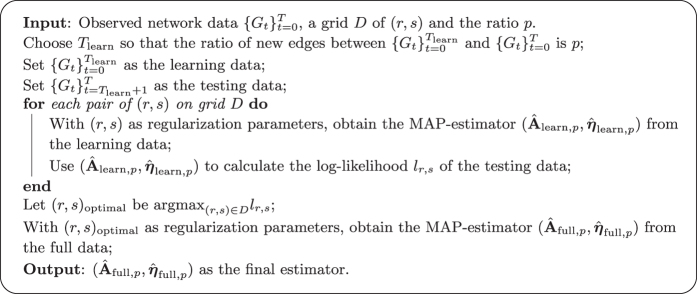
The workflow of PAFit. In this paper, we use *p* = 0.75. An example of the grid *D* is the one that is used in Fig. 3 with *r* in (0, 0.25, 0.5, 1, 2, 5, 10, 20) and *s* in (0.1, 0.5, 0.75, 1, 1.25, 1.5, 2, 5, 10). See the Discussion Section for more details on the choice of *p* and *D*.

**Figure 3:**
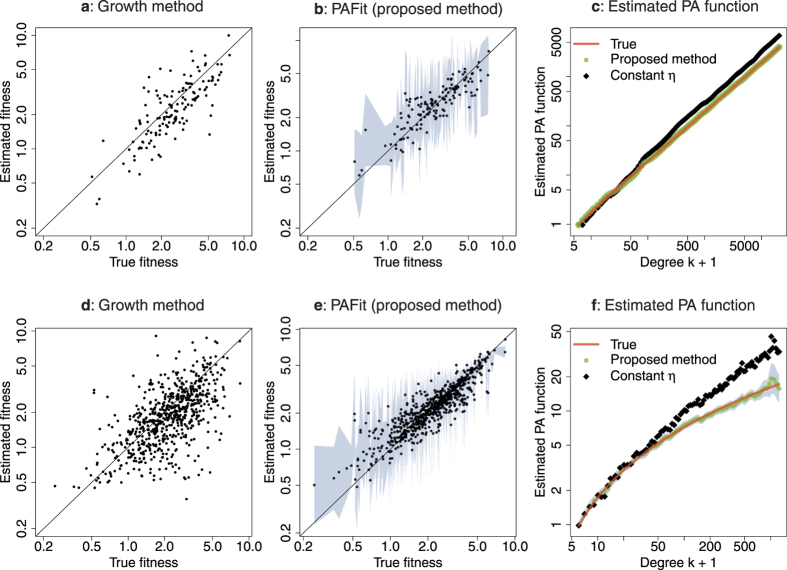
Estimation by PAFit (our proposal) and the growth method (Kong *et al*.^29^) in two simulated examples. First row: *A*_*k*_ = max(*k*, 1). Second row: *A*_*k*_ = 3(log max(*k*, 1))^2^ + 1. The true underlying node fitnesses are sampled from a gamma distribution with mean 1 and variance 1/s* = 1. The plots are on a log-log scale. Since the number of logarithmic bins for the PA function is *B* = 100 in both examples, there are 100 points in each estimated PA function. The lightblue band around the estimated values represents two-sigma confidence intervals of these estimated values. “Constant *η*” is our name for the case when we assume the Krapivsky *et al*. model^24^, and use PAFit with all node fitness *η* fixed at 1 to estimate only the PA function. In these two examples, PAFit successfully recovers the PA function and node fitnesses simultaneously, as well as outperforms existing methods.

In each example, we follow the workflow of PAFit shown in Fig. 2 over a grid *D* with *r* in (0, 0.25, 0.5, 1, 2, 5, 10, 20) and *s* in (0.1, 0.5, 0.75, 1, 1.25, 1.5, 2, 5, 10). For the *A*_*k*_ = max(*k*, 1) example, the optimal combination is (*r, s*) = (5, 2). For the *A*_*k*_ = 3(log max(*k*, 1))^2^ + 1 example, the optimal one is (*r, s*) = (0.25, 2). The final estimators are shown in Fig. 3b,c,e,f.

Let us first consider the results of the growth method shown in Fig. 3a,d. In the case of the linear PA function, the growth method gave *e*_*η*_ = 0.16 and *r*_*η*_ = 0.74. For the non-log-linear PA function *A*_*k*_ = 3(log max(*k*, 1))^2^ + 1, the growth method gave *e*_*η*_ = 0.26 and *r*_*η*_ = 0.57. It is encouraging to note that the growth method performed better in the linear case, which is precisely the situation for which it is designed. Although the growth method performed acceptably well in both cases, one can see that the estimated fitness does not follow the true fitness closely, especially when *A*_*k*_ is non-log-linear.

Turning our attention to the results PAFit shown in Fig. 3b,c,e,f, it gave *e*_*η*_ = 0.08, *r*_*η*_ = 0.84, *e*_*A*_ = 0.0007 when *A*_*k*_ is linear; and *e*_*η*_ = 0.09, *r*_*η*_ = 0.9, *e*_*A*_ = 0.004 when *A*_*k*_ = 3(log max(*k*, 1))^2^ + 1. We can see that PAFit succeeded in the simultaneous recovery of *A*_*k*_ and *η*_*i*_ in both cases, and clearly outperformed the growth method. We note that one advantage of PAFit is that it can naturally estimate confidence intervals for the estimated results.

To find out whether joint estimation of PA and fitness is needed in estimating the PA function, we compare PAFit with a method we named “constant *η*”, in which we also use PAFit, but assume the model of Krapivsky *et al*.^24^ with *η* fixed at 1. The constant *η* method gave *e*_*A*_ = 0.003 when *A*_*k*_ is linear, and *e*_*A*_ = 0.04 when *A*_*k*_ is non-log-linear. These two numbers, which are much worse than those of the simultaneous estimation results (*e*_*A*_ = 0.0007 when *A*_*k*_ is linear and *e*_*A*_ = 0.004 when *A*_*k*_ is non-log-linear), clearly indicate the need for simultaneous estimation of PA and fitness.

We note that for the PAFit method there is a tendency such that the more new edges a node acquires in the growth process, the better its fitness can be estimated. The simple reason for this is that the number of new edges a node acquires corresponds to the amount of observed data for that node.

We make some remarks about the chosen values of *r* and *s*. The chosen *r* correctly reflects the fact that it is the regularization parameter that enforces the log-linear form *k*^*α*^. In the log-linear example, the chosen *r* is large (*r* = 5), while in the non-log-linear example, the chosen *r* is small (*r* = 0.25). Although PAFit did not recover the true parameter s* of the underlying gamma distribution of node fitnesses, we note that in both examples the chosen *s*’s are very close to s*. Due to random fluctuations, s* does not necessarily best represent the observed data. Indeed, the estimated PA functions and node fitnesses in both examples agree well with the true values. In Supplementary Information Section S1.1, we give more examples of choosing the regularization parameters in six simulated networks, and show that in all cases PAFit succeeds in recovering both PA and fitness simultaneously.

In these two simulated examples, the true distribution of node fitnesses is the same as the prior distribution of node fitnesses in PAFit, i.e., both are gamma distributions. On the other hand, the growth method is a distribution-free method. In Supplementary Information Section S1.2, we show four examples where PAFit outperforms the growth method when the true distribution of node fitnesses is log-normal or power-law, which are more heavy-tailed than the gamma distribution.

Finally, in Supplementary Section S1.3, we perform a simulation study with 48 combinations of different s* and different true functional forms of *A*_*k*_, where each combination consists of 100 simulated networks. We show that PAFit generally outperforms existing methods in estimating PA and node fitness.

### Real-world dataset

We apply PAFit to a real-world network: a directed multiple network representing wall-posts between a subset of Facebook users from 2005 to 2009^54^. A directed edge in the network represents a post from one user to another user’s wall. One might speculate that the following factors are important for a user to attract posts to his/her wall: a) How much information about his/her life that he/she publicises: his/her birthday, engagement, promotion, etc. b) How influential and/or authoritative his/her own posts are which call for further discussions from other people; and c) how responsive the user is in responding to existing wall posts. We then can hypothesize fitness *η*_*i*_ to be a combination of these three factors averaged over time. On the other hand, PA can be interpreted as a herding effect of some kind: *A*_*k*_ captures the averaged pattern of how people will post more on a wall based solely on how many wall post it already has, regardless of all other factors such as the wall owner’s characteristics, the content of existing posts, and so on.

We choose the network at the onset of year 2007 as the initial network, and use the data added from 2007 to 2009 to estimate *A*_*k*_ and *η*_*i*_. We also grouped the edges into daily time-steps as has previously been done in other social network datasets^56^,^57^. The total number of nodes |*V*| and total number of edges |*E*| in the final snapshot of the network are 46952 and 876993, respectively. Meanwhile, *T* = 754 is the number of observed time-steps, while Δ|*V*| = 37967 and Δ|*E*| = 803930 are the increments of nodes and edges after time-step *t* = 0, respectively. We fit the power-law distribution *k*^−*γ*^ to the in-degree distribution of the final snapshot by the MLE method^26^. We choose 40 as the starting degree from which the distribution is assumed to be power-law, and find the estimated *γ* to be 2.3. *K* = 1428 is the maximum degree that appears in the growth process. Finally, we use *B* = 50 logarithmic bins for the PA function.

### Co-existence of PA and fitness

**Figure 4:**
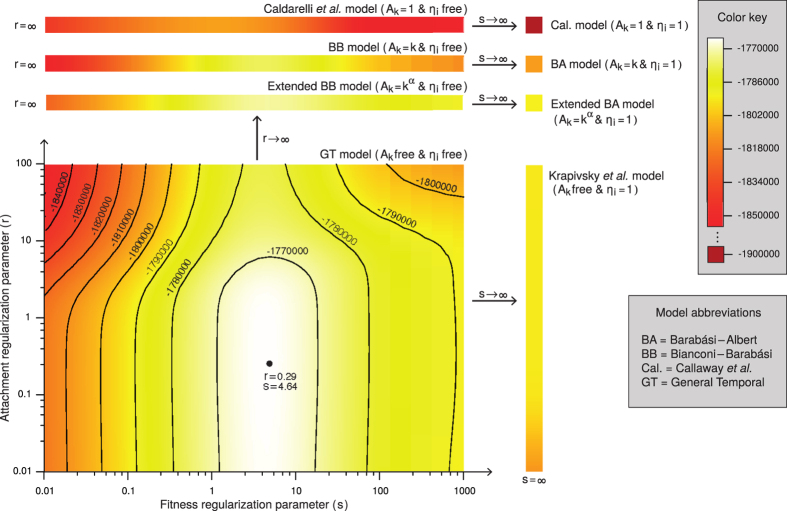
Density model landscape. For a two-dimensional grid of the regularization parameters *r* and *s*, which includes all models in Table 2, we learn *A*_*k*_ and *η*_*i*_ using the learning data, and plot the log-likelihood of the unseen testing data. The relation of the models are shown clearly. The log-likelihood at the peak (*r, s*) = (0.29, 4.64) is −1765188. The log-likelihood given by growth method (which assumes the BB model) is −1936320, which is lower than the minimum value in the figure.

We found that for the Facebook dataset, the optimum combination of the regularization parameters is when (*r, s*) = (0.29, 4.64). As can be seen from the density plot in Fig. 4, this point is well inside the area of the GT model. This indicates the necessity of simultaneous estimation of both fitness and PA free of any assumptions. Estimating either *η*_*i*_ or *A*_*k*_ in isolation, or estimating the attachment exponent *α* and node fitness *η*_*i*_ jointly with the assumption *A*_*k*_ = *k*^*α*^ as in the extended BB model gave much worse log-likelihood of the testing data than the best combination.

**Figure 5:**
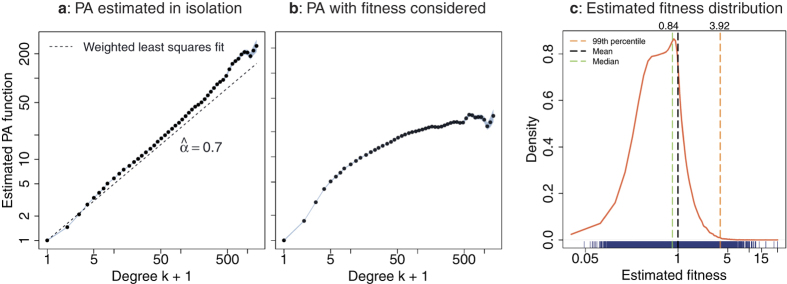
Co-existence of “rich-get-richer” and “fit-get-richer” effects in the Facebook wall-post network. (**a**) Estimated *A*_*k*_ with constant node fitness (existing method^31^). *A*_*k*_ is reasonably log-linear (i.e. in the form *k*^*α*^) when fitness is ignored. (**b**) Estimated *A*_*k*_ when node fitness is taken into account using our proposed method. The functional form of *A*_*k*_ becomes highly non-log-linear. Since this case is the optimal one that best represents the dataset, it shows the need for joint estimation of PA and node fitness, as well as the need for considering PA functions that deviate from the popular form *k*^*α*^. (**c**) The distribution of estimated fitnesses. The horizontal axis is in log-scale with the blue marginal rugs indicating the individual node fitnesses. The distribution is heavy-tailed, which suggests the existence of the “fit-get-richer” effect.

Figure 5a shows the estimated *A*_*k*_ when fitness is ignored, while Fig. 5b,c show the estimated *A*_*k*_ and the distribution *P*(*η*) of the estimated *η*_*i*_ in the case of joint estimation, respectively. We also ran PAFit for a number of other combinations of *r* and *s* around the maximum point (0.29, 4.64), as well as for a number of different values of *r* when *s* is held fixed at 4.64. We found that the estimation results in these cases are similar to the estimation results when we use the best combination (figures not shown). This indicates, understandably, that our method is robust. We also note that, reassuringly, with the optimum combination of parameters, the estimation results of *A*_*k*_ and *η*_*i*_ when using only the learning data are similar the estimation results when we use the full dataset (see Supplementary Information Section S1.4 and Supplementary Fig. S6). This assures us that the growth mechanisms of the network in the learning data and in the full data are reasonably similar. This implies that the use of the learning data and the testing data to choose the regularization parameters as in our aforementioned procedure is sound. We also note that the main findings in this section do not change if we change the ratio between learning data and full data from 0.75 to 0.5 or 0.9 (see Supplementary Information Section S1.4).

Inspecting the estimated *A*_*k*_ in Fig. 5b, we observe several important findings. Firstly, the estimated *A*_*k*_ is an increasing function, thus clearly signals the existence of the rich-get-richer phenomenon (corresponding to an increasing *A*_*k*_ on average). Secondly, the estimated *A*_*k*_ is highly non-linear in log-scale, which is different from the widely assumed log-linear model *A*_*k*_ = *k*^*α*^. This reinforces the need to consider non-log-linear functional forms when modelling the PA function^31^,^47^. Since the estimated *A*_*k*_ is nearly log-linear when fitness is ignored (Fig. 5a), this dataset shows the need for joint estimation of PA and node fitness. Finally, the form of the PA function gradually becomes flat when the degree is large, which might indicate a limit in our capacity to make new acquaintances or new collaborations^55^.

To get a sense of the growth rate of the estimated PA function in comparison with the conventional log-linear form, we fitted the function *A*_*k*_ = *k*^*α*^ to the estimated *A*_*k*_ by a weighted least squares method where the weights are inversely proportional to the estimated variance of the 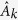
31. Using this procedure, we found that 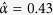, which implies that the PA function is sub-linear in this dataset. Finally, comparing with the estimated PA function in the case of constant node fitness in Fig. 5a, the estimated PA function in Fig. 5b became lower. This indicates that the rich-get-richer effect became weaker when the fit-get-richer effect was taken into account, which is expected since a portion of a node’s ability to attract new edges could then be explained by its fitness.

Turning our attention to the estimated node fitness in Fig. 5c, while almost all node fitnesses are concentrated around the mean, which is 1, there are some nodes with very high fitness. This highly non-uniformity of the fitness distribution is a clear signal of the fit-get-richer phenomenon.

### Fitness dominates PA

**Figure 6:**
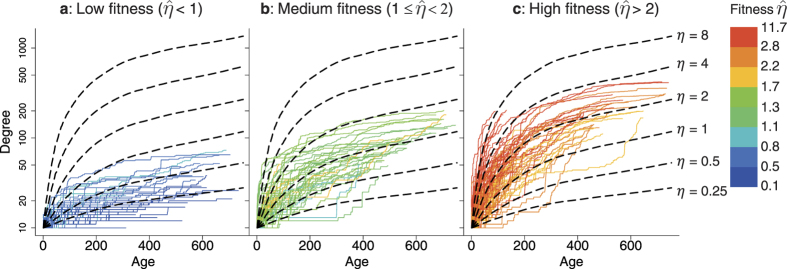
High fitness nodes have dominant degree growth curves. Node age is defined as the time since it first attains degree 10. The dashed lines are theoretical growth curves of a generic node with true fitness *η* = 8, 4, 2, 1, 0.5 and 0.25, based on the GT model. These theoretical curves are added as visual guides, and are calculated using the procedure described in Supplementary Information Section S1.6. Overall, the fit-get-richer effect is visible: nodes with high fitness have steep growth curves, while nodes with low fitness have more moderate ones. The real-world curves agree well with the theoretical curves, which indicates that the estimation results of PAFit are consistent with the GT model. (**a**) 200 randomly chosen curves from nodes whose 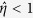. Most of these curves broadly follow around the *η* = 0.25 and *η* = 0.5 theoretical curves. Some real curves with darker blue color are well below the *η* = 0.25 theoretical curve, while some real curves with lighter blue color rise near the *η* = 1 theoretical curve. (**b**) 200 randomly chosen curves from nodes whose 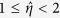. Most of these real curves are between the *η* = 1 and *η* = 2 theoretical curves. (**c**) 200 randomly chosen curves from nodes whose 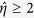. While most of these real curves are between the *η* = 2 and *η* = 4 theoretical curves, some very steep real curves rise around the *η* = 8 theoretical curve.

Now with evidence decisively pointing to the co-existence of rich-get-richer and fit-get-richer phenomena, one cannot help but ask the question as to exactly which one of the two effects played the greater role in governing the evolution of node degree over the growth of the network. To investigate the relation between fitness and the degree of a node, in Fig. 6a–c, we drew the degree growth curves of 200 random chosen nodes from three groups: low-fitness nodes with 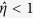, medium-fitness nodes with 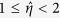 and high-fitness nodes with 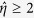, respectively. We also plot theoretical degree growth curves of a generic node with fitness *η* = 8, 4, 2, 1, 0.5 and 0.25 to serve as anchors (see Supplementary Information Section S1.6 for the way to calculate these curves).

In Fig. 6, the degree of a high fitness node tends to grow faster than that of a low fitness node. This results in a general trend: curves in Fig. 6a mostly have a near-horizontal orientation, while those in Fig. 6b have mild upward slopes, and most of those in Fig. 6c have steep slopes. These observations indicates clearly the fit-get-richer effect. We also note that the real-world data curves generally agree well with the theoretical curves, which implies that the estimated fitness of PAFit is consistent with the underlying GT model. We perform some additional analyses on the degree growth curves in Supplementary Information Section S1.5.

**Figure 7:**
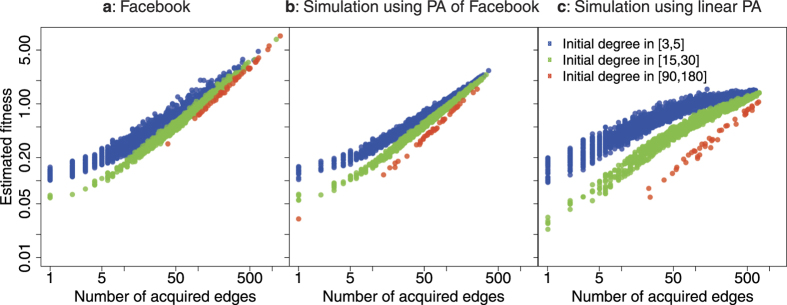
Fitness plays the major role in deciding the number of future edges that a given node acquires. (**a**) Facebook dataset. We consider three groups of nodes with initial degree *k*_0_ in [3, 5], [15, 30] and [90, 180]. The differences in numbers of acquired edges are largly explained by fitness, not by initial degree. (**b**) A simulated network using the estimated PA function of Facebook as the true PA function. (**c**) Another simulated network with the much stronger *A*_*k*_ = *k* as the true PA function. This time, the three groups are well separated by their colors.

To further investigate the intertwined effects of the PA function and node fitness, in Fig. 7 we plot the number of acquired new edges of a node versus its estimated fitness for three groups of nodes with different initial degrees (degree at time 0). We found that in the Facebook dataset, fitness plays the major role in deciding the number of edges a node acquired. In Fig. 7a, the difference in the number of new edges a node acquired is largely explained by its fitness. While the initial degree, and hence the PA function, does have a visible effect, its effect is small, since the three groups overlap substantially. A plausible explanation for this phenomenon is that, in the Facebook dataset, the estimated PA function is rather weak (as mentioned earlier, the estimated attachment exponent *α* is about 0.43). For checking this explanation, we generate two simulated networks as controlled experiments. In both simulations, we set the initial network *G*_0_, the number of new edges and new nodes at each time-step the same as what were observed in the Facebook dataset. We also use the variance of Facebook’s estimated fitness (Fig. 5c) for the variance of the gamma distribution to generate true node fitness. On the one hand, Fig. 7b shows the situation when we use the same estimated PA function of the Facebook dataset (Fig. 5b). We can spot a similarity with Fig. 7a: the number of new edges of a node is largely explained by its fitness, not by its initial degree. On the other hand, in Fig. 7c we show the plot when we use the much stronger PA functional form *A*_*k*_ = *k*. This time the three groups are clearly separated by their initial degrees. This shows how the situation would look like if a strong PA function dominated fitness. These two simulated examples strongly imply that a weak PA function is the reason for the dominance of fitness in the Facebook dataset.

## Discussion

We have proposed a statistically sound Bayesian method, called PAFit, for estimating both the PA function (*A*_*k*_) and node fitness (*η*_*i*_) in growing complex networks. PAFit is nonparametric in the sense that it does not fix any particular functional form for either *A*_*k*_ or *η*_*i*_, so that it is able to detect different types of functional forms.

PAFit uses a PA regularization term and a fitness regularization term to avoid overfitting. The fitness regularization term is equivalent to placing a gamma prior distribution on each fitness. There is the question of how well PAFit performs when the true distribution differs from a gamma distribution. Although an extensive study involving different types of true fitness distributions is needed to answer this question, as a first step, we show by four simulated examples that our method performs well even when the true fitness distribution follows a power-law or log-normal form, which is more heavy-tailed than the gamma distribution.

We use the likelihood of the testing data for choosing the PAFit regularization parameters. Some well-known statistical criterions such as the Bayesian Information Criterion or the Bayes factor are not known to be applicable in our situation, since not only the data here is not independent and identically distributed, but the number of parameters in our model (**A** and *η*) is also a random variable that grows with the size of the network and the number of time-steps. This differers from a standard statistical setting. While the risk of overfitting still remains in PAFit, we contend that our method serves as an important first step before more involved statistical procedures can be developed for our model.

We reported clear evidence for the joint presence of the “rich-get-richer” phenomenon (corresponding to an increasing *A*_*k*_ on average) and the fit-get-richer phenomenon in a Facebook wall-post network. The functional form of the PA function *A*_*k*_ differs from the conventional log-linear form, *A*_*k*_ = *k*^*α*^. We also observed that the distribution of node fitnesses is heavy-tailed with a number of nodes having very high fitness.

We found that in the Facebook wall-post network, fitness plays the major role in deciding the number of future edges a node acquires, while the PA function has comparatively little effect. We caution that our analysis of the roles of PA and fitness here is rather qualitative. For a more conclusive answer, it might be needed to develop a quantitative method to measure the contribution of PA and fitness.

In this paper, we set the ratio *p* between the learning data and the full data to be 0.75. Although this choice seems to be arbitrary, we showed that the results in the Facebook dataset do not change if we use *p* = 0.5 or *p* = 0.9. As discussed in the Methods Section, given the bias-variance trade-off in choosing *p*, we contend that our choice of *p* = 0.75 in PAFit represents a reasonable balance between two extremes of this trade-off.

Although the above contributions are established entirely in the setting of growing networks with time-invariant PA and node fitness functions, one potential merit of our estimated *A*_*k*_ and *η*_*i*_ is that, since they can be interpreted as the time-averaged version of some time-varying *A*_*k*_(*t*) and *η*_*i*_(*t*), they are arguably more robust to the network fluctuations, as well as the changes in the number of new edges *m*(*t*) and new nodes *n*(*t*) at each time-step. At a minimum our method stands as a first step towards the full resolution of the estimation of time-dependent *A*_*k*_(*t*) and *η*_*i*_(*t*).

Our method requires a grid *D* to search for the optimal pair of *r* and *s*. As can be seen from Fig. 4, the log-likelihood of the testing data has only one peak, and gradually changes only on log-scale. We also reported that the final estimator 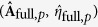 would almost not change if we used different (*r, s*) around the optimal pair. We note that we have the same observations on simulated networks. Thus for the initial probing, we recommend to use a coarse grid on logarithmic scale in order to quickly cover a large range. Then one might use another logarithmic scale grid around the peak of the previous search for local exploring.

There are various directions for future research. First, given the new findings we obtained, it is only natural to conduct a large-scale application of PAFit to public data to discover the extent to which our findings in the Facebook wall-post dataset generalizes to other complex networks. Secondly, convergence of the PAFit method, as well as consistency and asymptotic normality of the MLE, are open research questions. Thirdly, there are some immediate extensions of the PAFit framework worth pursuing. For example, since PAFit assumes the time-invariant case of a growing network, it would be interesting to see if one can extend the methodology to the time-varying case with not only addition, but also deletion of nodes and edges. Another interesting extension is to use more heavy-tailed distributions such as the log-normal or power-law as prior distributions for node fitness. Finally, the PAFit method assumes that we fully observed the sequence 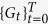 of network snapshots. However, there are situations where we can only observe the final network snapshot, namely *G*_*T*_, but none of the preceding snapshots. Making PAFit able to jointly estimate the PA function and node fitness will enable us to ask the core question of co-existence of rich-get-richer and fit-get-richer, as well as all other questions concerning the functional forms of the PA function and node fitness, for these networks too.

## Methods

### The General Temporal model

The PAFit method assumes the GT model, which is a generative network model for both directed and undirected growing networks^31^. According to the GT model, a network is generated by starting from some seed network *G*_0_, then at each time-step *t* ≥ 1, *m*(*t*) new edges and *n*(*t*) new nodes are added to *G*_*t*−1_ to form *G*_*t*_. Note that *m*(*t*) may consist of both new edges that emanate from the *n*(*t*) new nodes and emergent new edges between existing nodes. This allows wide applications of PAFit in real-world situations, where new edges do emerge between existing nodes.

Here we state the GT model for directed networks. The details of the undirected GT model is provided in Supplementary Information Section S2.2. When a new edge is added to the network *G*_*t*−1_, it will connect to an existing node *v*_*i*_ with probability


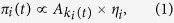


where *k*_*i*_(*t*) is the in-degree of node *v*_*i*_ at the onset of time *t*. For a directed network, given *m*(*t*) and *n*(*t*), Eq. (1) does not completely determine *G*_*t*_, since it ignores the source nodes of the edges. But the quantities *A*_*k*_ and *η*_*i*_ are by definition concerned with the ability of nodes to acquire new edges, and thus are independent of the out-degrees of the source nodes in the directed case. Therefore, modelling only the destination node as in Eq. (1) is actually enough for the estimation of *A*_*k*_ and *η*_*i*_. The GT model includes a number of important generative network models as special cases, as can be seen from Table 1.

Finally, it is important to note that, although the GT model in this paper contains only the addition of nodes and edges, this is purely for simplicity and clarity of exposition. The PAFit method is easily extendable to handle the case when there are deletions, as long as the probabilistic mechanism of deletions is independent of the addition mechanism, and does not involve *A*_*k*_ and *η*_*i*_.

### Bayesian estimation

Here we provide a brief discussion of the Bayesian estimation for the directed GT model. The case of the undirected GT model is treated in a similar way. The full details of both cases are described in the Supplementary Information Sections S2.3 and S2.4. Our observed data is the sequence 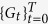 of networks. Let *K* and *N* be the maximum degree and the final number of nodes in a GT model network, respectively. Let 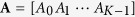 and 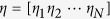 be the parameter vectors we want to estimate.

Adopting a Bayesian approach, PAFit maximizes the following objective function:


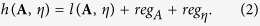


*l*(**A**, *η*) is the log-likelihood function of the data:


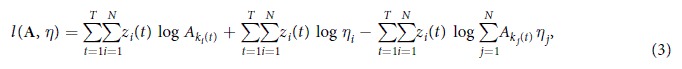


with *z*_*i*_(*t*) be the number of new edges that connect to node *v*_*i*_ at the onset of time *t. reg*_*A*_ is the following regularization term for the PA function:


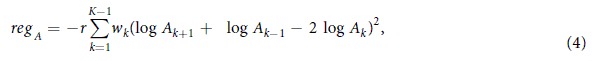


with 
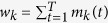
 and *m*_*k*_(*t*) is the number of edges that connect to a degree *k* node at time *t. reg*_*η*_ is the following regularization term for node fitness:


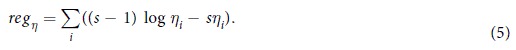


These two regularization terms are equivalent to Bayesian prior distributions for *A*_*k*_ and *η*_*i*_. Thus *r* of *reg*_*A*_ and *s* of *reg*_*η*_ are hyper-parameters in the Bayesian interpretation, and the estimated (*A*_*k*_, *η*_*i*_) is the MAP estimate.

By using *reg*_*A*_ in Eq. (4), we estimate *A*_*k*_ without any assumptions on its functional form, but will be able to fall back to the widely-assumed functional form *A*_*k*_ = *k*^*α*^ when needed, since this regularization term becomes approximately 0 when *A*_*k*_ = *k*^*α*^, and is negative otherwise. Note that in order to balance the strength of the regularization and the observed data, each quadratic term in Eq. (4) is then weighted by the number of observed data points *w*_*k*_ of degree *k*. If *r* is 0, then we estimate the PA function without any prior assumptions. The larger the value of *r*, the more the form of the estimated *A*_*k*_ approaches *k*^*α*^. When *r* = ∞, the strength of Eq. (4) overwhelms the observed data, and forces *A*_*k*_ to be *k*^*α*^. We note that the regularization term in Eq. (4) is the same as in ref. 31.

We derive this regularization term as follows. Starting from *A*_*k*_ = *k*^*α*^, for non-zero log *k* this is equivalent to log *A*_*k*_/log *k* = *α*. Now using the same formula but with *k* replaced by *k* + 1 and *k* − 1 yields log *A*_*k*+1_/log(*k* + 1) = *α* and log *A*_*k*−1_/log(*k* − 1) = *α*. This implies log *A*_*k*+1_/log(*k* + 1) − log *A*_*k*_/log *k* = log *A*_*k*_/log *k* − log *A*_*k*−1_/log(*k* − 1). This is equivalent to log *A*_*k*+1_/log(*k* + 1) + log *A*_*k*−1_/log(*k* − 1) − 2 log *A*_*k*_/log *k* = 0. For moderately large *k*, since log(*k* + 1) ≈ log(*k* − 1) ≈ log *k*, the last equation leads to log *A*_*k*+1_ + log *A*_*k*−1_ − 2 log *A*_*k*_ = 0, whose left hand side forms the quadratic terms of Eq. (4).

For node fitness, the regularization term *reg*_*η*_ has the same effect as placing a gamma prior with shape and rate parameters *s* on each *η*_*i*_, since it is the logarithm of the density function of the gamma distribution. This prior setting is viable, given that the *η*_*i*_’s are positive real-numbers. Gamma priors have been used extensively for the rating parameters of the Plackett-Luce model, whose likelihood function consists of multinomial probabilities just as our GT model^58^,^59^,^60^. In the context of growing complex networks, we contend that only the gamma distribution has been explored as a fitness prior^46^. So in this paper we follow convention and adopt a gamma prior. We note that in large datasets, the likelihood is likely to dominate the prior’s information, so a different prior setting for node fitness is unlikely to change the numerical result significantly.

The mean and variance of our gamma prior are 1 and 1/*s*, respectively. Thus the larger the value of *s*, the smaller the variance of the node fitness. In the limiting case when *s* = ∞, all the *η*_*i*_’s take the value 1. Thus *s* = ∞ is effectively equivalent to the case when we fix all *η*_*i*_ at 1 and only estimate *A*_*k*_, i.e. the Krapivsky *et al*. model in Table 1.

The objective function in Eq. (2) can be efficiently maximized by a Minorize-Maximization (MM) algorithm^49^, which in this case is also known as a ConCave-Convex Procedure^61^. Starting from some initial value (**A**^(0)^, *η*^(0)^) at iteration *q* = 0, the proposed algorithm iteratively calculates (**A**^(*q*+1)^, *η*^(*q*+1)^) from (**A**^(*q*)^, *η*^(*q*)^), until some convergence condition (such as the relative difference between successive values of the objective function reaches some threshold) is met. At each iteration *q*, the proposed algorithm decomposes the multi-variate maximization problem into many one-dimensional problems in a way such that the value of *h*(*η*, **A**) is guaranteed to increase after each iteration. The one-dimensionality of these sub-problems allow them to be solved efficiently in parallel. We implemented the algorithm in the R package PAFit^52^.

Lastly, although we use *A*_*k*_’s in all equations and algorithms in this paper for ease of exposition, in practice one invariably needs to perform binning on the degrees for more reliable results. In binning, *A*_*k*_’s are set to be *ω*_*i*_ for all *k* in the *i*-th bin, then *ω*_1_, 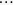, *ω*_*B*_ are taken as parameters to be estimated. Here *B* is the number of bins. All the equations and algorithms described in this paper are valid with *A*_*k*_’s replaced by *ω*_*i*_’s. The number of *k*’s inside a bin is determined by that bin’s width. In PAFit, we choose logarithmic binning in order to create small-width bins in low degree regions, where we have many data points for each degree, and large-width bins in the region of high-degrees, where we have few data points for each degree^31^. In our experience, 20 to 200 is a good range for the number of bins, *B*.

### Choosing regularization parameters by testing data

Here we give more details on the workflow shown in Fig. 2. In this paper, we use 0.75 as the value for *p*, the ratio of number of new edges between the learning data and the full data. In other words, *T*_learn_, the final time-step in the learning data, is chosen so that 
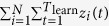
 is approximately three times of 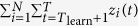. Here recall that *z*_*i*_(*t*) is the number of new edges that connect to node *v*_*i*_ at the onset of time *t*. When we calculate the log-likelihood of the testing data, we use Eq. (3) but with the set {1, …, *N*} restricted to the set of nodes that appeared in the learning data, since we do not have *η*_*i*_ for the nodes *v*_*i*_ that newly appear in the testing data.

We note here about the inherent bias-variance trade-off in choosing *p*, the ratio between the learning data and the full data. When *p* is large, the bias of 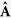 and 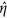 is small, but the variance is large. To understand this statement let us take an example when *p* = 0.99. In this case, our estimated *A*_*k*_ and *η*_*i*_ using only the learning data are very close to those when we use the full data, since almost all of the full data is learning data. This means the bias is small. But since the testing data, which is the remaining one percent of the full data, has so few observations, any small random fluctuation can greatly change the optimal pair of *r* and *s*, and thus change 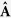 and 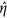. This means the variance is big. When *p* is small, a reverse situation occurs: the variance is small, but the bias is large.

While we do not have a theoretical reason to support our choice of *p* = 0.75 in this paper, we argue that this value of *p* represents a reasonable balance between the two extremes of bias-variance trade-off. On the one hand, Supplementary Fig. S6 suggests that there is a sense of convergence of the result when *p* approaches 1: the estimated results when *p* = 0.75 and *p* = 0.9 are very similar, and thus the choice of *p* is not sensitive in this region. On the other hand, the same figure also shows that *p* = 0.5 is too small to get a reliable result.

It is important to stress that the above approach not only provides a statistically sound way to determine the regularization parameters *r* and *s*, but also answers the fundamental question: which of the models in Table 1 best describes the evolving process of a network? To answer this question, we fit each of the models in Table 1 to the learning dataset, and evaluate their log-likelihoods on the testing dataset.

## Additional Information

**How to cite this article**: Pham, T. *et al*. Joint estimation of preferential attachment and node fitness in growing complex networks. *Sci. Rep.* **6**, 32558; doi: 10.1038/srep32558 (2016).

## Supplementary Material

Supplementary Information

## Figures and Tables

**Figure 1:**
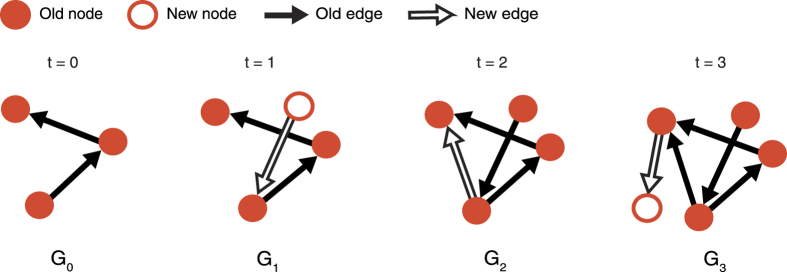
An example of a growing network. At each time-step, new nodes and edges are added to the network. The number of new nodes and edges at each time-step are free to vary. Note that new edges may emanate from and connect to any old or new nodes. Some examples are: a new edge from a new node to an old node (the network at *t* = 1), a new edge between existing nodes (the network at *t* = 2), and a new edge from an existing node to a new node (the network at *t* = 3).

**Table 1:** Prominent generative network models that are included as special cases of the General Temporal (GT) model.

Generative Network Model	PA Function	Fitness	Reference
GT model	Free	Free	Pham *et al*.^31^,^47^
Callaway *et al*.	*A*_*k*_ = 1	*η*_*i*_ = 1	Callaway *et al*.^33^
BA model	*A*_*k*_ = *k*	*η*_*i*_ = 1	Barabási and Albert^15^
Extended BA model	*A*_*k*_ = *k*^*α*^	*η*_*i*_ = 1	Krapivsky *et al*.^32^
Krapivsky *et al*.	Free	*η*_*i*_ = 1	Krapivsky *et al*.^24^
Caldarelli model	*A*_*k*_ = 1	Free	Caldarelli *et al*.^28^
BB model	*A*_*k*_ = *k*	Free	Bianconi and Barabási^16^
Extended BB model	*A*_*k*_ = *k*^*α*^	Free	Not previously considered.

The GT model is our model for growing network generation. In the model, *Pr*(Node *v*_*i*_ receives new links) ∝ *A*_*k*_ × *η*_*i*_ where both the PA function *A*_*k*_ and node fitnesses *η*_*i*_ are time-invariant. Callaway *et al*.’s model is reminiscent of the Erdös-Rényi (ER) model^48^ in so far as connections are formed uniformly at random at each time-step.

**Table 2:** Generative network models and the regularization parameters *r* and *s*.

Generative Network Model	r	s	Reference
GT model	Free	Free	Pham *et al*.^31^,^47^
Callaway *et al*.	∞	∞	Callaway *et al*.^33^
BA model	∞	∞	Barabási and Albert^15^
Extended BA model	∞	∞	Krapivsky *et al*.^32^
Krapivsky *et al*.	Free	∞	Krapivsky *et al*.^24^
Caldarelli model	∞	Free	Caldarelli *et al*.^28^
BB model	∞	Free	Bianconi and Barabási^16^
Extended BB model	∞	Free	Not previously considered.

The parameter *r* is the PA function regularization parameter and the parameter *s* is the fitness regularization parameter. The bigger the value of *r*, the more *A*_*k*_ assumes the form *k*^*α*^. Since *s* is inversely proportional to the variance of the gamma prior of node fitness, the bigger the value of *s*, the more the estimated node fitnesses concentrate around 1. By varying *r* and *s*, we can investigate many scenarios of the co-existence of PA and fitness (see the Methods Section for an explanation).
