# Supplementary material for: Joint estimation of preferential attachment and node fitness in growing complex networks
Source: Sci Rep. 2016 Sep 7;6:32558. doi: 10.1038/srep32558 (PMC5013469; doi:10.1038/srep32558)
Supplement: Supplementary Information [file srep32558-s1.pdf]

Joint estimation of preferential attachment and node fitness in growing complex networks Supplementary Information

Thong Pham [1,*], Paul Sheridan [2], and Hidetoshi Shimodaira[1]

[1]Division of Mathematical Science, Graduate School of Engineering Science, Osaka University, Osaka, Japan
[2]The Institute of Medical Science, The University of Tokyo, Tokyo, Japan
[*]thongpham@sigmath.es.osaka-u.ac.jp

S1 Supplementary Results

We provide here additional numerical results of PAFit on simulated examples and the Facebook wall-post dataset. In Section S1.1, we show six examples of choosing the regularization parameters r and s. Section S1.2 then shows estimation results in four simulated datasets when the true fitness distribution is not the gamma distribution. Section S1.3 provides an extensive Monte Carlo simulation investigating the performance of PAFit in comparison with existing methods. In Section S1.4, we discuss the estimation results in the Facebook dataset when varying the ratio p. Section S1.5 provides additional analyses on the Facebook dataset. Finally, Section S1.6 describes the method to calculate the theoretical degree growth curves.

S1.1 Examples of choosing the regularization parameters

We apply the workflow in Fig. 2 for six simulated networks. Each simulated network has a total of $N = 1000$ nodes. At each time-step, $m = 5$ new edges and $n = 1$ new node are added to the network. In all these networks, the true distribution of node fitnesses is a gamma distribution with mean 1 and variance $1/s^*$. We set the number of logarithmic bins B for the PA function to 50. The optimal regularization parameters (r, s) together with the estimated values of A_k and η_i are shown in Supplementary Fig. S1. Overall, PAFit recovers the true parameter s^* in most cases. In the case of the BB model when PAFit failed to do so, the estimated s is very close to s^*, and the estimated A_k and η_i are also very close to the true values.

S1.2 Examples of heavy-tailed distributions as true distributions for node fitness

Here we provide four examples when the true fitness distribution is not the gamma distribution. Starting from a seed network with 100 nodes, $m(t) = 5$ new edges and $n(t) = 1$ new node are added at each time-step t until the total number of nodes reached is $N = 10000$. We use two distributions for node fitness: a log-normal distribution with mean 0 and standard deviation 1 in log scale, and a power-law (Pareto) distribution with shape parameter 2.5 and scale parameter 0.6 (these parameters are chosen so that the mean is 1). Supplementary Fig. S2 shows these two distributions in comparison with the gamma distribution whose mean and variance are 1. We set the number of logarithmic bins B for the PA function to 100. Supplementary Fig. S3 shows the estimation results when the true distribution is log-normal, while Supplementary Fig. S4 shows the estimation results when the true distribution is power-law. PAFit outperforms existing methods in both cases.

S1.3 Overall performance of PAFit

The first aspect we want to investigate is the effects of different functional forms of the true A_k on the quality of the estimation of PAFit. For this purpose, we use three different functional forms. Supplementary Table S1 shows a summary of the true PA functions that we used. The first one is $A_k = \max(k, 1)^\alpha$, which is the widely assumed log-linear form. The second one is $A_k = \min(500, \max(k, 1))^\beta$. This functional form presents a realistic situation in which PA follows the log-linear form in the low degree region, then plateaus after some threshold degree k. Lastly, $A_k = 3\left(\log\max(k, 1)\right)^b + 1$ is an elaborate non-log-linear functional form. In terms of growth rates, all the PA functions we consider in this section are sub-linear, except, of course, the log-linear model for which $\alpha \geq 1$.

The other aspect we want to understand is the effect of the distribution of true fitnesses. To this end, we generate node fitnesses from three different distributions: gamma distributions with mean 1 and variance $1/s^*$ with $s^* = 0.1$, 1, and 10. Note that when $s^* = 1$, the gamma distribution reduces to the exponential distribution. The larger the value of s^*, the smaller the variance of the true fitness. In summary, there is a total of $16 \times 3 = 48$ different combinations of the true A_k and true distribution of η_i's.

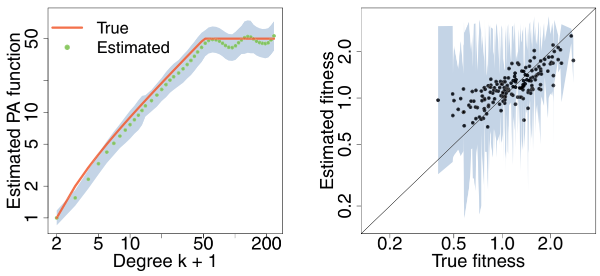

True: $A_k = \min(\max(k,1),50)$ and $s^* = 5$.
Chosen: $r = 3$ and $s = 5$.

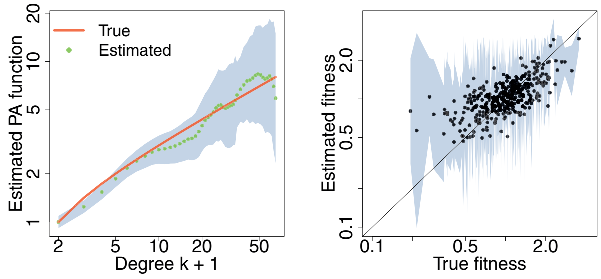

True: $A_k = \max(k,1)^{0.5}$ and $s^* = 5$.
Chosen: $r = 2$ and $s = 5$.

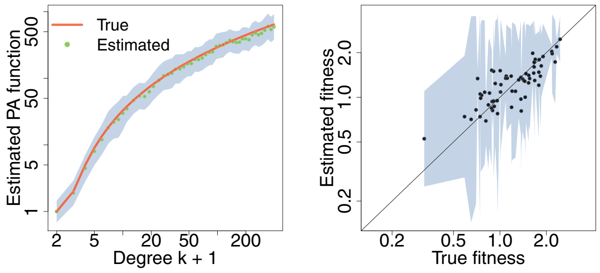

True: $A_k = 3(\log\max(k,1))^3 + 1$ and $s^* = 5$.
Chosen: $r = 0.1$ and $s = 5$.

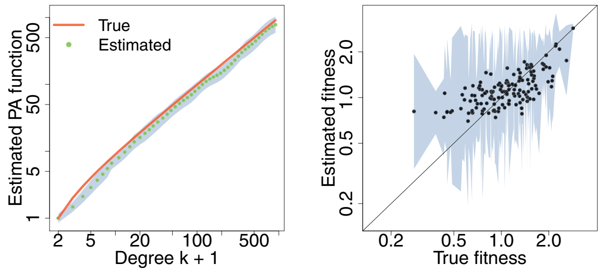

True: $A_k = \max(k,1)$ and $s^* = 5$ (BB model).
Chosen: $r = 5$ and $s = 6$.

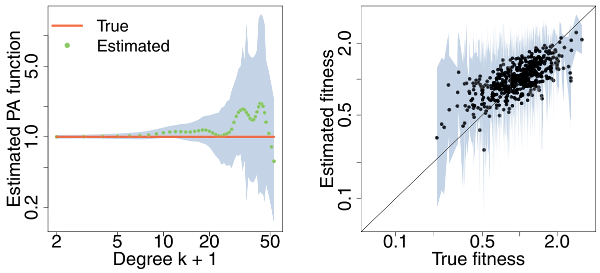

True: $A_k = 1$ and $s^* = 5$ (Caldarelli model) .
Chosen: $r = 5$ and $s = 5$.

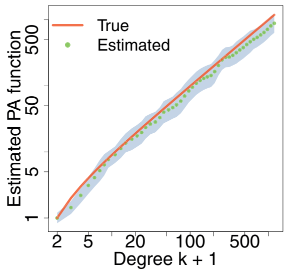

True: $A_k = \max(k,1)$ and $s^* = \infty$ (BA model).
Chosen: $r = 0.1$ and $s = \infty$.

Supplementary Figure S1. Some examples of choosing the optimum regularization parameters. In all these networks, the true distribution of node fitnesses is a gamma distribution with mean 1 and variance $1/s^*$. For each combination of r and s, A_k and η_i are estimated using the learning data, and then the log-likelihood of the unseen testing data is calculated. The optimal combination of r and s is the one that gives the highest log-likelihood of the unseen testing data. The lightblue band at estimated points represents the estimated two-sigma confidence intervals of the associated point estimates.

Next we specify the regularization parameters of A_k (the parameter r) and η_i (the parameter s). For the regularization of A_k, we set $r = 0.1$, as Pham *et al.*[2] have shown through simulations that a small amount of regularization helps reducing the error in estimating A_k. To investigate the effect of mis-specification of the true distribution of node fitnesses, we consider three different values for the regularization parameter s: 0.1, 1 and 10.

To summarize, the simulation settings are as follows. For each of the 48 combinations of true functions of A_k and true distributions of η_i, we generate 100 networks according to the GT model. Starting from a seed network with 20 nodes, at each

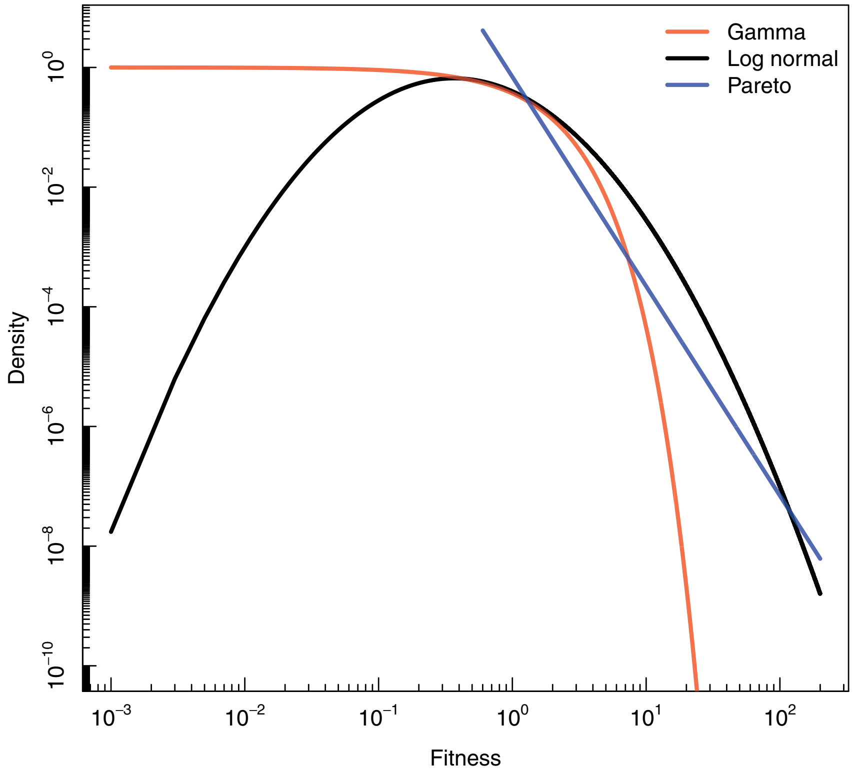

Supplementary Figure S2. Three fitness distributions used in simulation and their tails. The plot is on a log-log scale. Both the log-normal and the power-law (Pareto) distributions have a heavier right-tail than the gamma distribution.

time-step, $m(t) = 5$ new edges and $n(t) = 1$ new node were added, until a total of 5000 nodes is reached. We then apply PAFit to each generated network. Regarding η_i, we consider three cases: $s = 0.1$, 1 and 10. Regarding A_k, the parameter r is fixed at 0.1. The A_k are also grouped into logarithmic bins and the number of bins B is 20. Supplementary Fig. S5 shows average relative errors e_η and e_A averaged over 100 repetitions.

First consider e_η in estimating η_i, we see that PAFit outperforms the growth method, even when the regularization parameter s is mis-specified. The effect of mis-specifying s is clear, when the case s is equal to the true parameter s^* almost always gave the lowest error.

Next, let us consider the performance of PAFit through e_A. Since there are no existing methods that estimate A_k with considerations for node fitness, we have to introduce the following two variants of PAFit to be served as reasonable baselines. The first variant, called *baseline1*, estimates A_k while fixing the fitness of node v_i at the number of new edges it has acquired in the growth process, i.e. letting $\eta_i = \sum_t z_i(t)$. Since we can see PAFit markedly outperforms *baseline1* in all situations, the seemingly reasonable heuristic of using the number of acquired new edges as fitness results in surprisingly poor estimation. In the second variant called *baseline2*, we essentially ignored the effect of fitness by fixing $\eta_i = 1$ for each node v_i. Note that this is equivalent to the method proposed in our previous work[2]. PAFit outperforms *baseline2* in almost all cases except when $A_k = k^\alpha$ when α is near 1.5.

With such a large value of the attachment exponent, the winner-take-all effect becomes prominent even in finite data. As explained in the Introduction Section of the main text, the winner-take-all situation indicates a situation where only a handful of nodes, perhaps even only one node, actually acquire new edges, while the vast majority of nodes do not acquire any new edges at all. This winner-take-all effect drastically skews the amount of data for each node. Also note that, e_A generally increases with α in this model. This phenomenon, which has also been confirmed in the case of estimating A_k in isolation[2], indicates that the estimation problem gets "harder" as the degree of the winner-take-all effect increases. The simulated results suggest that fixing $\eta_i = 1$ for all i in such cases, which serves as a regularization that helps to reduce the dimension of the problem, actually

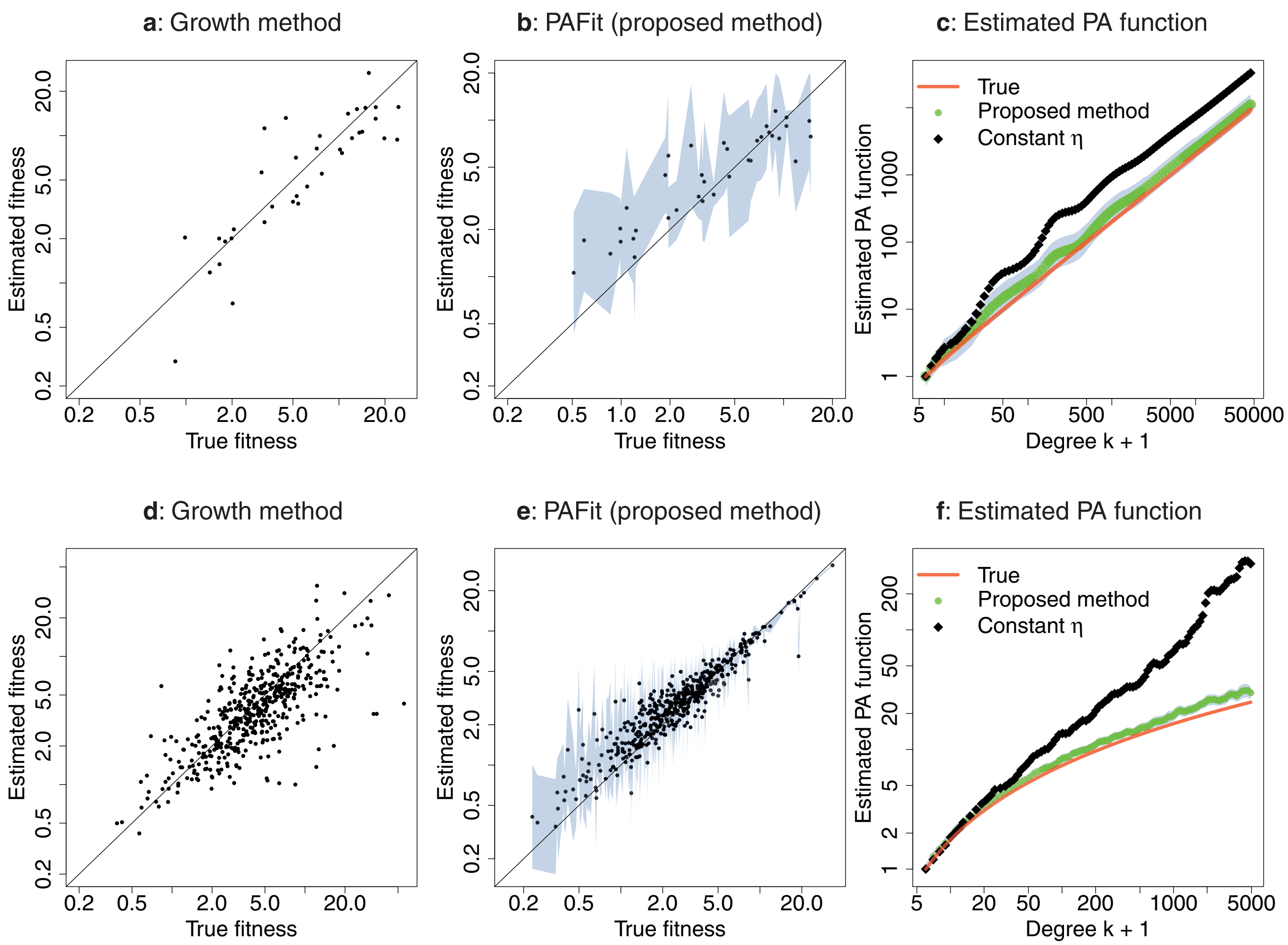

Supplementary Figure S3. Estimation by PAFit (our proposal) and the growth method (Kong *et al.*[1]) when the true node fitnesses are sampled from a log-normal distribution. First row: $A_k = \max(k, 1)$. Second row: $A_k = 3\left(\log \max(k, 1)\right)^2 + 1$. The plots are on a log-log scale. The lightblue band at estimated points represents the estimated two-sigma confidence intervals of the associated point estimates. For the first row, the growth method gave the correleration coefficient between estimated and true fitness $r_\eta = 0.74$ (the higher the correleration coefficient, the better the estimation) and the relative error $e_\eta = 0.27$ (the lower the relative error, the better the estimation). With the chosen (r, s) being $(5, 1)$, PAFit gave $r_\eta = 0.83$ and $e_\eta = 0.18$. For the second row, the growth method gave $r_\eta = 0.6$ and $e_\eta = 0.26$. With the chosen (r, s) being $(0.25, 1.25)$, PAFit gave $r_\eta = 0.96$ and $e_\eta = 0.11$. To conclude, PAFit outperformed the growth method in both examples.

results in better estimation of A_k.

The effects of mis-specifying s on estimating A_k is surprising: the value $s = 0.1$ gives better estimation of A_k than other values of s, even when the true parameter s^* is not 0.1. Note that in the log-linear model with $\alpha \geq 1$, the specification of s seems to be unimportant, since all values of s gave almost the same error.

Overall, we can conclude that PAFit can indeed estimate both A_k and η_i successfully. PAFit also performs better than the growth method in estimating η_i. The specification of s seems to be important, but when the mis-specification is not so great (as in this example, about 10 times), the estimated results of both η_i and A_k do not worsen much.

S1.4 Estimation results when varying the ratio between learning data and full data

While we have used $p = 0.75$ in the main text, here we present estimation results for two additional cases when the ratio p between the learning data and the full data is 0.5 and 0.9. When $p = 0.5$, the optimal combination of the regularization parameters is $(r, s) = (0.28, 32.47)$, while this combination is $(r, s) = (0.03, 2.66)$ when $p = 0.9$. When the ratio between the

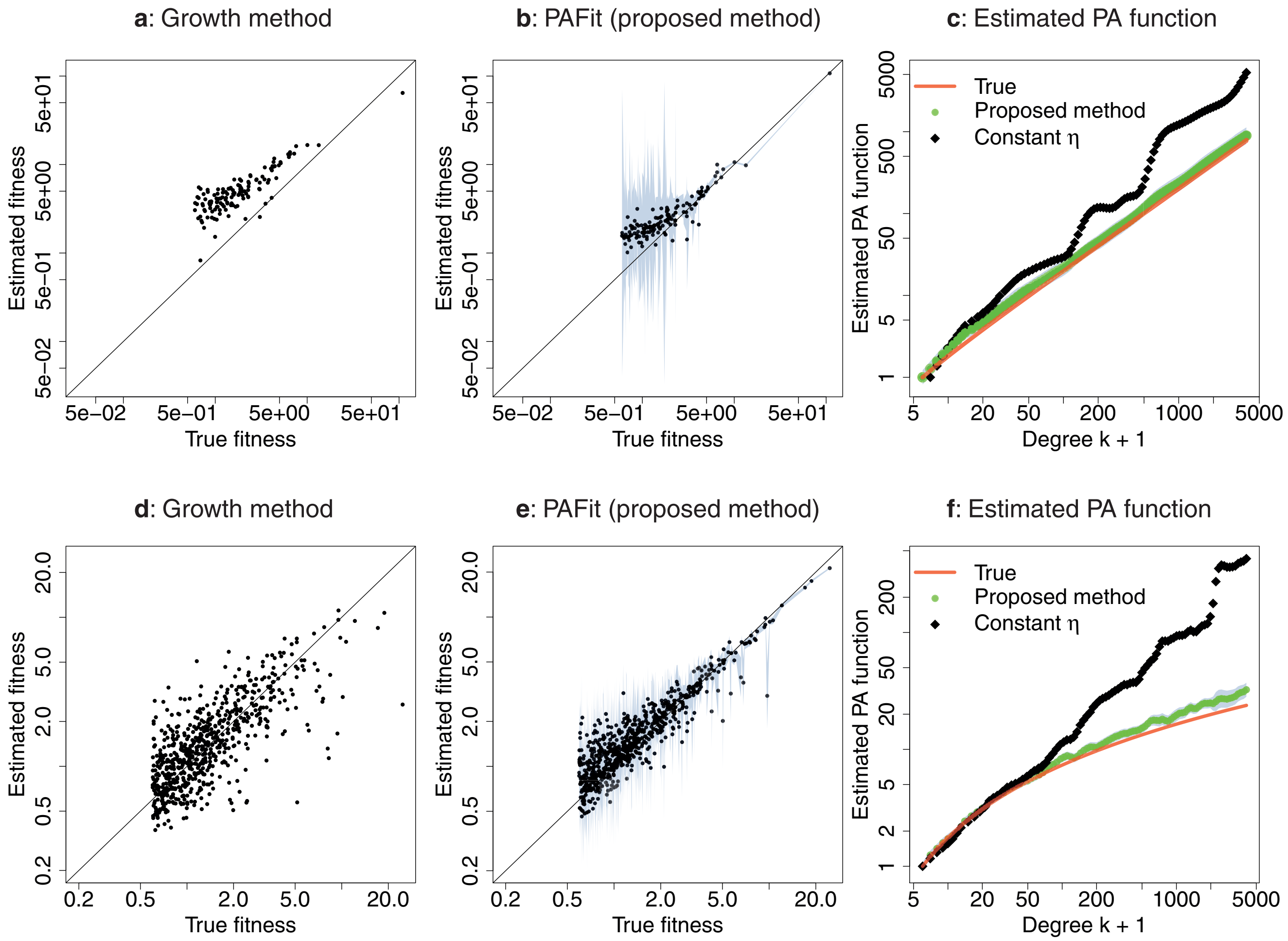

Supplementary Figure S4. Estimation by PAFit (our proposal) and the growth method (Kong *et al.*[1]) when the true node fitnesses are sampled from a Pareto distribution. First row: $A_k = \max(k,1)$. Second row: $A_k = 3\left(\log\max(k,1)\right)^2 + 1$. The plots are on a log-log scale. The lightblue band at estimated points represents the estimated two-sigma confidence intervals of the associated point estimates. For the first row, the growth method gave the correleration coefficient between estimated and true fitness $r_\eta = 0.93$ (the higher the correleration coefficient, the better the estimation) and the relative error $e_\eta = 0.16$ (the lower the relative error, the better the estimation). With the chosen (r,s) being $(20,0.4)$, PAFit gave $r_\eta = 0.99$ and $e_\eta = 0.12$. For the second row, the growth method gave $r_\eta = 0.69$ and $e_\eta = 0.19$. With the chosen (r,s) being $(0.25,1.25)$, PAFit gave $r_\eta = 0.96$ and $e_\eta = 0.08$. To conclude, PAFit outperformed the growth method in both examples.

learning data and the full data is p, recall that $\hat{\mathbf{A}}_{\text{full},p}$ and $\hat{\eta}_{\text{full},p}$ are the estimated PA function and the estimated fitness using the full data with the optimal (r,s), respectively (see Fig. 2). Similarly, here we let $\hat{\mathbf{A}}_{\text{learn},p}$ be the estimated PA function using the learning data with the same optimal (r,s). Supplementary Fig. S6a shows $\hat{\mathbf{A}}_{\text{learn},p}$ and Supplementary Fig. S6b shows $\hat{\mathbf{A}}_{\text{full},p}$ for three different values of p. Supplementary Fig. S7 shows $\hat{\eta}_{\text{full},0.75}$ in comparison with $\hat{\eta}_{\text{full},0.5}$ and $\hat{\eta}_{\text{full},0.9}$ for nodes whose number of acquired edges is at least 50.

Overall, the results concerning the PA function do not change significantly if we use $p = 0.9$ or $p = 0.5$. In Supplementary Fig. S6, we notice that $\hat{\mathbf{A}}_{\text{learn},0.75}$ and $\hat{\mathbf{A}}_{\text{full},0.75}$ are reasonably similar, and they are both strikingly close to both $\hat{\mathbf{A}}_{\text{learn},0.9}$ and $\hat{\mathbf{A}}_{\text{full},0.9}$. Since we use $\hat{\mathbf{A}}_{\text{full},p}$ for subsequent analyses, the closeness of $\hat{\mathbf{A}}_{\text{full},0.75}$ and $\hat{\mathbf{A}}_{\text{full},0.9}$ indicates there is almost no change if we choose $p = 0.9$. While $\hat{\mathbf{A}}_{\text{learn},0.5}$ is substantially different from $\hat{\mathbf{A}}_{\text{full},0.5}$, $\hat{A}_{\text{full},0.5}$ are reassuringly close to $\mathbf{A}_{\text{full},0.75}$. This closeness again implies that the results concerning the PA function does not change significantly even if $p = 0.5$.

The results concerning node fitness also do not change significantly if we use $p = 0.9$ or $p = 0.5$. In Supplementary Fig. S7, the point $(\hat{\eta}_{\text{full},0.75}, \hat{\eta}_{\text{full},0.9})$ are strikingly close to the $y = x$ line. The R^2 coefficient, whose values close to 1 often indicate

PA function	Parameter	Value
$A_k = \max(k,1)^{\alpha}$	α	0.5,0.6,...,1.5 (11 values)
$A_k = \min(500, \max(k,1))^{\beta}$	β	0.8, 1.0, 1.2
$A_k = 3\left(\log\max(k,1)\right)^{b} + 1$	b	2, 3

Supplementary Table S1. Summary of true PA functions used in Monte Carlo simulations. There is a total of 16 different functions.

a linear relationship, is 0.99 in this case. Although the points $(\hat{\eta}_{\text{full},0.75}, \hat{\eta}_{\text{full},0.5})$ deviate a bit from the $y = x$ line , the R^2 coefficient is 0.91, which can still be considered high.

S1.5 Additional data analyses in the Facebook wall-post dataset

We show in Supplementary Fig. S8 the same degree growth curves as in Fig. 6, but this time with the horizontal axis being time. The same trend in which nodes with high fitness have steep growth curves is still visible here, but is difficult to see due to the differences in birth times of the curves. Nevertheless this figure reveals some additional information that cannot be seen in Fig. 6.

One might notice a sudden increase in the number of new nodes at each time-step from around time-step 600. This increase can be seen more clearly from the change in the slope of the number of new nodes $n(t)$ in Supplementary Fig. S9a. The increase of new nodes leads to an increase of the number of node fitnesses we have to estimate. One might worry that there might be not enough data to do this adequately.

Fortunately, since this increase in $n(t)$ is accompanied by an increase in the number of new edges $m(t)$ (Supplementary Fig. S9b), the normalized number of new edges is stable (Supplementary Fig. S9c), and thus the whole system is in fact stable. The normalized number of new edges, which is defined as $\mu(t) = m(t)/\left(\sum_{j:v_j \text{ exists at } t} A_{k_j(t)}\eta_j\right)$, can be intuitively viewed as the amount of new data available at time-step t, after taking into consideration the differences in network sizes or degree distributions, etc. at different time-steps. $\mu(t)$ is calculated using the observed $k_i(t)$, while in Supplementary Methods Section S1.6 it was calculated using the expected $k_i(t)$.

We note that the oscillations observed in $\mu(t)$ are caused by the oscillations in $m(t)$. The normalized number of new edges $\mu(t)$, by definition, can be affected by $m(t)$ and $\sum_{j:v_j \text{ exists at } t} A_{k_j(t)}\eta_j$, and the latter in turn can be affected by $n(t)$. But while there is no periodicity in $n(t)$ (Supplementary Fig. S9d), a weekly period can be seen clearly in both the autocorrelation function of $m(t)$ (Supplementary Fig. S9e) and $\mu(t)$ (Supplementary Fig. S9f). We found that the cause of this periodicity is that $m(t)$, the number of new wall-posts, tends to increase toward the middle of the week and decrease on weekends (Supplementary Fig. S10).

Although Eq. (S1) implies that we can only compare the growth rate of two curves in Supplementary Fig. S8 at the same time-step, it is possible to use $\mu(t)$ to compare the growth rate of two degree growth curves of some nodes v_1 and v_2 (v_1 and v_2 can be the same) at two different time-steps t_1 and t_2. Assuming η_1 and η_2 are the fitness of v_1 and v_2 respectively, the corresponding growth rates of the two curves are $A_{k_1(t_1)} \times \mu(t_1) \times \eta_1$ and $A_{k_2(t_2)} \times \mu(t_2) \times \eta_2$. Furthermore, if $k_1(t_1) = k_2(t_2)$, i.e. the curve of v_1 at time t_1 is on the same horizontal level with the curve of v_2 at time t_2 in Supplementary Fig. S8, then we can ignore the PA factor when comparing them, and the two (relative) growth rates reduce to $\mu(t_1) \times \eta_1$ and $\mu(t_2) \times \eta_2$.

S1.6 Calculating expected degree growth curves

In short, the theoretical degree growth curves in Fig. 6 are expected degree growth curves calculated by evolving the whole network from the initial time-step using the estimated PA function (Fig. 5b) and estimated fitness of all real nodes (Fig. 5c), with the number of new edges and new nodes are kept exactly the same as in the observed evolution process. Specifically, for each $\eta = 8, 4, 2, 1, 0.5$ and 0.25, we repeat the following procedure:

- Step 0: We include a generic "ghost" node v_{ghost} with fitness η into the network at the initial time with initial degree $k_{\text{ghost}}(0) = 10$. Set $t = 0$.

- Step 1: If $t = T - 1$, go to Step 3. Otherwise, for every node v_i that appears in the network at time t we calculate

$$k_i(t+1) \leftarrow k_i(t) + \mu(t) \times A_{k_i(t)} \times \eta_i, \tag{S1}$$

  with $\mu(t) = \dfrac{m(t)}{\sum_{j:v_j \text{ exists at } t} A_{k_j(t)}\eta_j}$. With $m(t)$ be the number of new edges at time t, $\mu(t)$ can be interpreted as the normalized number of new edges at time t. $k_i(t)$ calculated this way is the expected value of the degree of node v_i at time-step t.

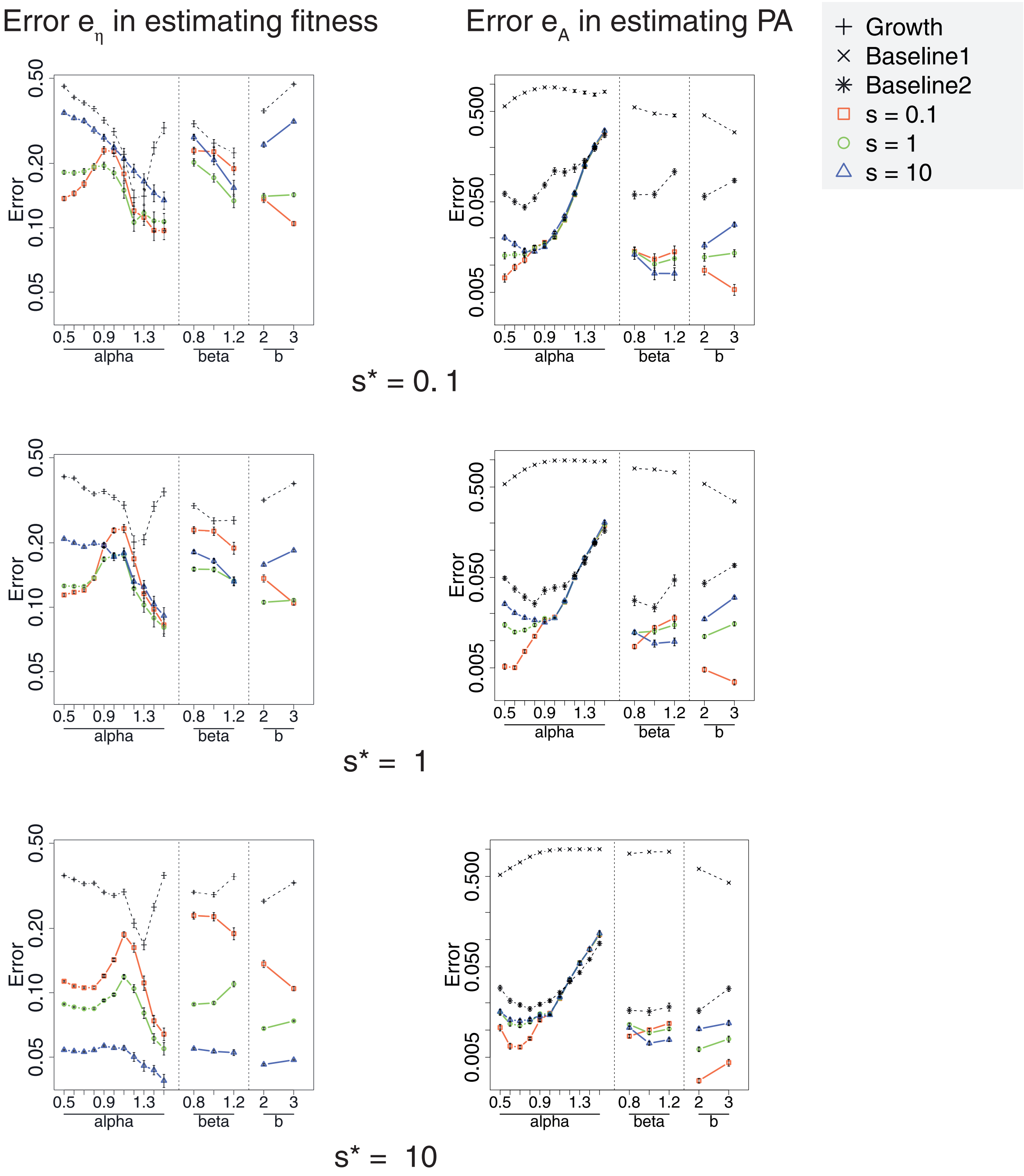

Supplementary Figure S5. **Results of Monte Carlo simulations.** Node fitnesses are generated from the a gamma distribution with mean 1 and variance $1/s^*$. The parameter s is the regularization parameter for fitness using in the algorithm. For each setting, we plot the mean error of each method from 100 simulated runs. The bars indicate the standard errors of the means.

- Step 2: Let $t \leftarrow t+1$ and back to Step 1.

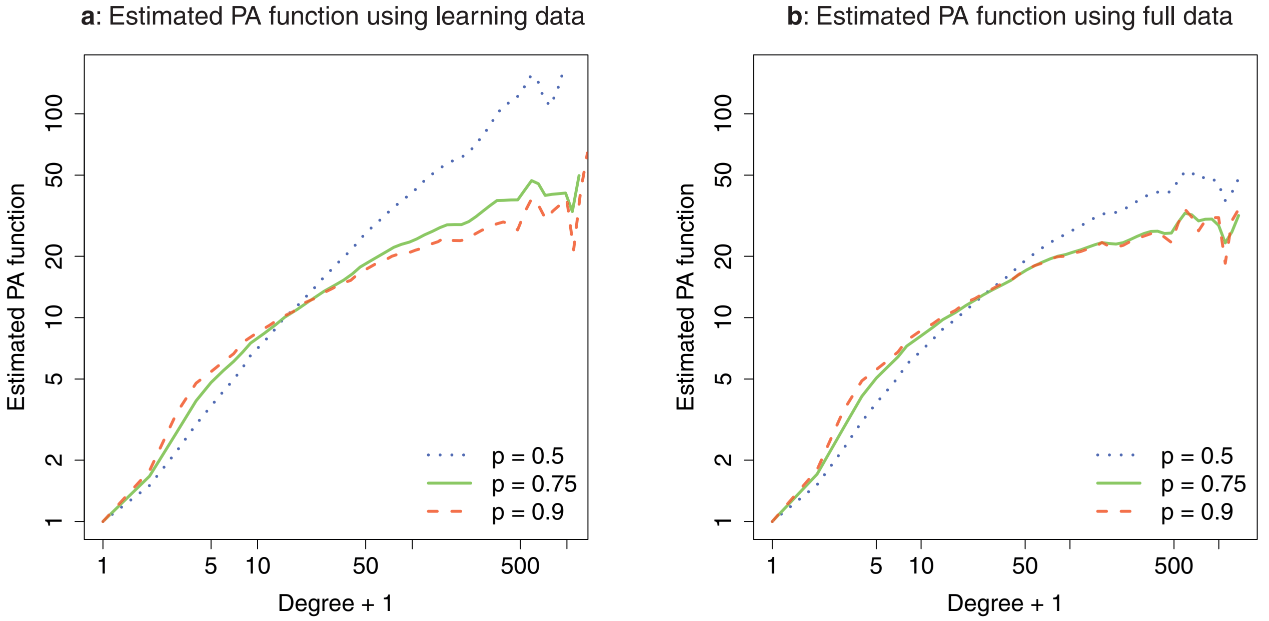

Supplementary Figure S6. Estimated PA functions in the Facebook wall-post dataset using the learning data ($\hat{\mathbf{A}}_{\mathbf{learn},p}$) and the full data ($\hat{\mathbf{A}}_{\mathbf{learn},p}$) for three cases when p, the ratio between learning data and full data, is 0.5, 0.75 **and** 0.9**.** The closeness of $\hat{\mathbf{A}}_{\text{full},0.75}$, $\hat{\mathbf{A}}_{\text{full},0.9}$ and $\hat{\mathbf{A}}_{\text{full},0.5}$ implies that the results concerning the PA function in the main text does not change significantly if $p = 0.9$ or $p = 0.5$.

- Step 3: Output the series $\{k_{\text{ghost}}(t), t = 0, \cdots, T-1\}$ as the theoretical growth curve of a node with fitness η.

S2 Supplementary Methods

In Section S2.1, we discuss related work on estimating PA and node fitness. We provide the definition of the undirected GT model in Section S2.2. The derivation of the log likelihood function is shown in Section S2.3. Finally, Section S2.4 provides the MM algorithm for estimation.

S2.1 Related work

Here we discuss existing work that is related to our proposed method. First consider the case when either A_k or η_i does not change with time, which is the situation we assume in this paper. In this case, the problem of estimating the PA function A_k while fixing η_i equal to 1 for all i has attracted the attention of many researchers, and there are a number of estimation methods[2–9]. While most of these methods assume the log-linear form $A_k = k^\alpha$, Pham *et al.*[2] recently provided a statistical method that does not assume any functional form for A_k. Assuming linear PA ($A_k = k$) upfront, Zhang *et al.*[10] proposed a method to find the optimal weight to combine the PA and clustering mechanisms. Middendorf *et al.*[11] used discriminative classification techniques to classify an evolving network according to one of the many network evolving mechanisms, including the linear PA mechanism. For the problem of estimating fitness in the time-invariant case, Kong *et al.*'s growth method[1] is the only existing method we know of that estimates η_i, albeit under the assumption that $A_k = k$. Their method estimates η_i by using the following asymptotic formula related to the degree of a node with its fitness:

$$\log k_i(t) = \eta_i \log t. \tag{S2}$$

The slope of the least-squares fit of $\log t$ to $\log k_i(t)$ gives us the fitness η_i. The fact that the growth method is an asymptotic method imposes several limits on the resulting estimation. Firstly, we have no assurances of how the method will behave on finite data. For example, the slope of the least-squares line of best-fit in Eq. (S2) might very well be negative, and thus lead to a nonsensical estimated value for η_i. Secondly, the assumptions needed to derive the asymptotic formula can be wrong in real-world networks. Most strikingly, the linear A_k assumption might not be supported by the observed data, as shown in the main text. From another different view point, Blasio *et al.*[12] considers the problem of distinguishing the effects of PA and fitness through statistical tests. They assumed that the PA effect only works in the linear form $A_k = k$, and did not consider the problem of estimating fitness.

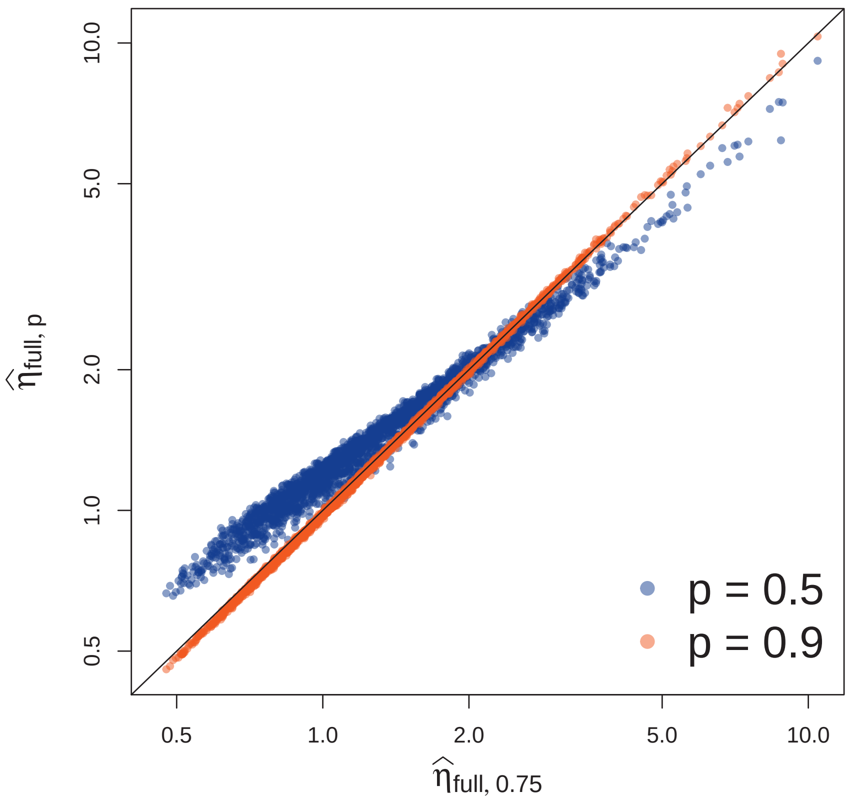

Supplementary Figure S7. Estimated fitness in the Facebook wall-post dataset using the full data, for three cases when p, the ratio between learning data and full data, is 0.5, 0.75 and 0.9. $\hat{\eta}_{\text{full},p}$ is the estimated fitness using the full data when the ratio between the learning data and the full data is p. $p = 0.75$ is the value used for the results in the main text. The three cases give similar fitness values.

If we allow time-variation in either the PA function or node fitness, there is a growing body of methods for estimating A_k or η_i. But comparing with the focus of our paper, the main focus of most of these estimation methods has been on a narrower class of complex networks, namely citation networks, where the effects of time on the probability that a paper or a patent will be cited are substantially studied and modeled. The time effect in these methods is also aptly called *aging* to describe the widely-used model in which $A_k(t)$ or $\eta_i(t)$ gradually decrease as the time t increases[13–15]. For the estimation of A_k with aging, various methods have been proposed[16–18]. All of them assume that $\eta_i = 1$ for all i. For estimating η_i with aging, some notable methods[19,20] are provided in the context of citation networks. Note again that the PA effect in these methods is assumed to be linear.

Lastly, generalizations of the ER random network model have long been proposed to model static networks. Since static networks are without growth, PA does not enter the picture, and the mechanism driving the birth of new edges is node fitness. For example, in the β- model[21], or p_1-model as it was originally called[22], the probability of an edge is proportional to the product of the fitness of its two end nodes. This model is closely related to the well-known Bradley-Terry model[23], which is often used to model pair comparisons. Statistical aspects of these models, including estimation methods and asymptotic properties, have been studied extensively[21,24–27]. From another related direction, asymptotic properties of some important statistics, such as the maximum degree K, of the BA model have also been researched rigorously[28,29]. All these works might be important references when we want to investigate asymptotic properties of the PAFit method.

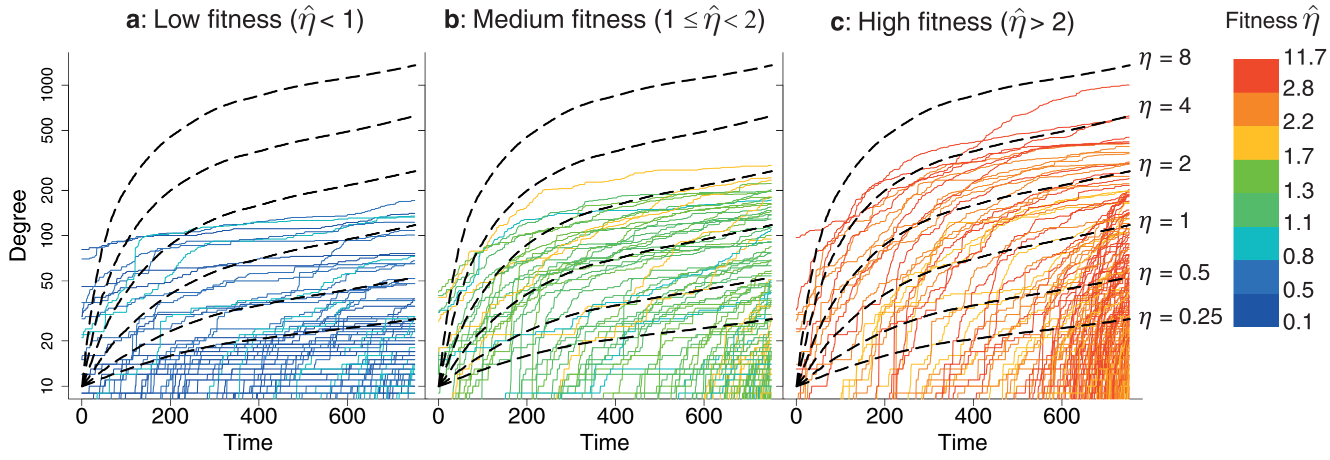

Supplementary Figure S8. Degree growth curves in Fig. 6 with time as horizontal axis. The dashed lines are theoretical growth curves of a generic node with true fitness $\eta = 8, 4, 2, 1, 0.5$ and 0.25, based on the GT model. These curves are added as visual guides, and are calculated using the procedure described in Supplementary Information Section S1.6. Nodes with high fitness can still be seen to have dominating growth curves. Using this figure, we can compare the growth rate of curves at the same time-step. Growth rates of curves at two different time-steps can also be compared if we use the information from $\mu(t)$, the normalized number of new edges. **a**: same curves as in Fig. 6a (200 randomly chosen curves from nodes whose $\hat{\eta} < 1$). **b**: same curves as in Fig. 6b (200 randomly chosen curves from nodes whose $1 \leq \hat{\eta} < 2$). **c**: same curves as in Fig. 6c (200 randomly chosen curves from nodes whose $\hat{\eta} \geq 2$).

S2.2 The undirected GT model

For an undirected network, the GT model defines the probability $\pi_{i,j}(t)$ of a new edge $e_{i,j}$ as follows.

$$\pi_{i,j}(t) \propto 2 \times A_{k_i(t)} \times \eta_i \times A_{k_j(t)} \times \eta_j, \tag{S3}$$

for $i \neq j$, and

$$\pi_{i,i}(t) \propto \left(A_{k_i(t)} \times \eta_i\right)^2, \tag{S4}$$

with $k_i(t)$ and $k_j(t)$ are degrees of nodes v_i and v_j at time-step t.

S2.3 Derivation of the log-likelihood function

Suppose that we observe the sequence $\{G_t\}_{t=0}^{T}$ of networks. Let K and N be the maximum degree and the final number of nodes in a GT model network, respectively. Let $\mathbf{A} = [A_0\, A_1 \cdots A_{K-1}]$ and $\eta = [\eta_1\ \eta_2\ \cdots\ \eta_N]$ be the parameter vectors we want to estimate.

The likelihood of the whole dataset can be built up sequentially from the likelihood of data at each time-step. For the initial graph G_0, we assume that its distribution is governed by some parameter vector θ_* that does not involve $\mathbf{A}$ and η. For example, if G_0 is an Erdös-Rényi random graph in which the numbers of nodes and edges both follow a Poisson distribution with mean 1, then θ_* is the vector $[1\ 1]$. Conditioning on G_{t-1} $(t \geq 1)$, the probability $P(G_t|G_{t-1})$ is the product of two terms: $P(m(t), n(t)|G_{t-1}, \theta(t))$ and $P(G_t|G_{t-1}, m(t), n(t), \mathbf{A}, \eta)$. The first term is the probability of the numbers of new edges and new nodes given G_{t-1}, which is assumed to be governed by a parameter vector $\theta(t)$ that does not involve $\mathbf{A}$ and η. The vector $\theta(t)$ can be the mean parameters of Poisson distributions, for example. The second term is the probability of how the new edges and new nodes form G_t given G_{t-1}, $m(t)$ and $n(t)$.

To summarize, the log-likelihood function of the whole dataset is

$$l(\mathbf{A}, \eta, \{\theta(t)\}_{t\geq 1}, \theta_*) = \sum_{t=1}^{T} \log P(G_t|G_{t-1}, m(t), n(t), \mathbf{A}, \eta) + \sum_{t=1}^{T} \log P(m(t), n(t)|G_{t-1}, \theta(t)) + \log P(G_0|\theta_*). \tag{S5}$$

Since θ_* and $\theta(t)$ do not involve $\mathbf{A}$ and η by assumption, we can ignore all but the first term on the right hand side of Eq. (S5) in deriving the MLE of $\mathbf{A}$ and η. We denote this term by $l(\mathbf{A}, \eta)$, which means the log-likelihood of the data with respect to $\mathbf{A}$ and η.

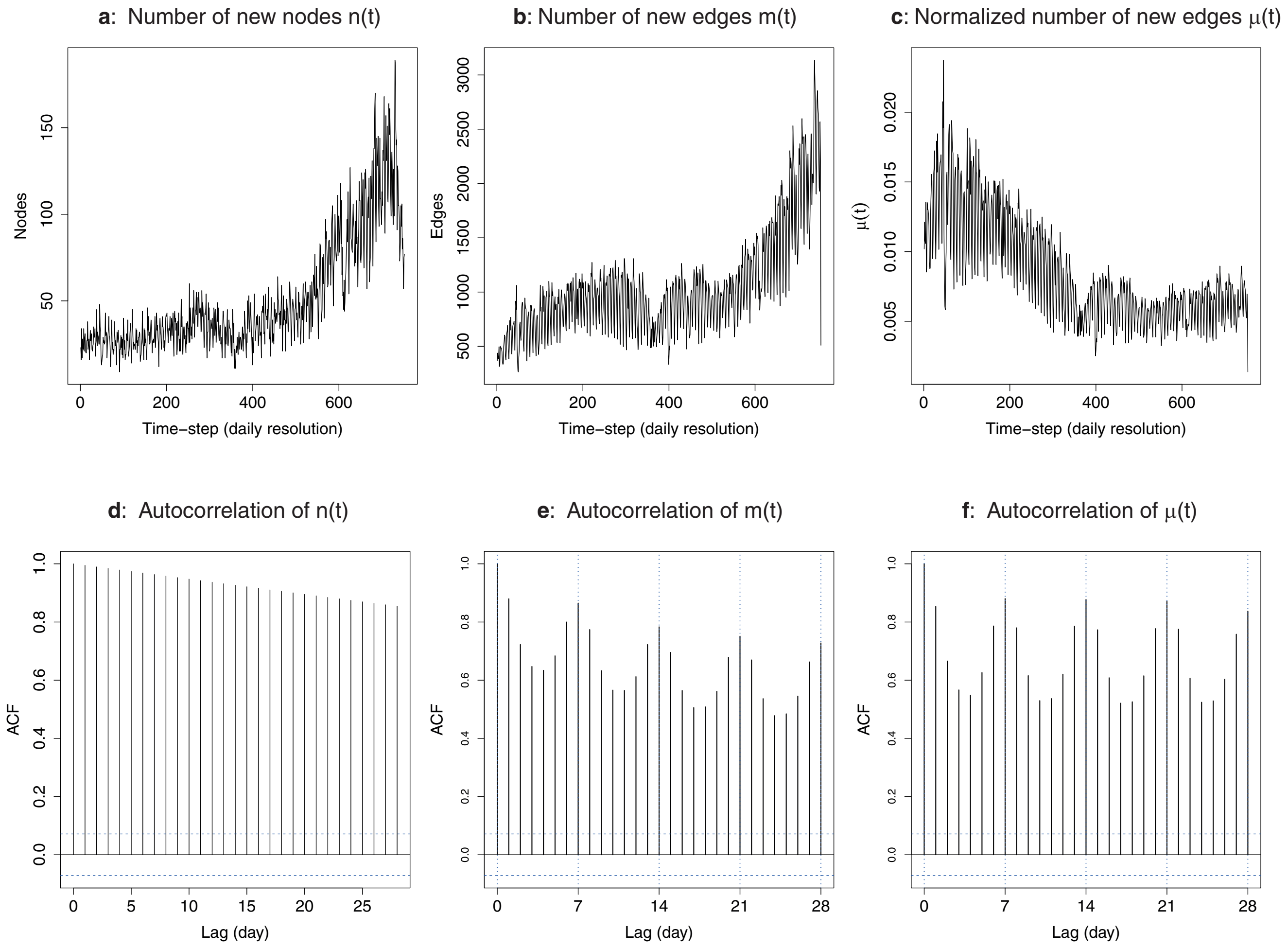

Supplementary Figure S9. Normalized number of new edges is stable and has a weekly period. **a**: Number of new nodes $n(t)$. There is a sudden increase from around time-step 600. **b**: Number of new edges $m(t)$. The increase in $n(t)$ is accompanied by an increase in $m(t)$. **c**: The normalized number of new edges $\mu(t)$. This $\mu(t)$, which can be intuitively viewed as the amount of new data available at each time-step, is actually quite stable in the region around time-step 600. **d**: Autocorrelation function of $n(t)$. There is no periodicity here. **e**: Autocorrelation function of $m(t)$. There is a clear weekly period. **f**: Autocorrelation function of $\mu(t)$. There is also a clear weekly period. Thus the periodicity of $\mu(t)$ is caused by the periodicity of $m(t)$, which in turn is caused by the tendency of $m(t)$ to increase toward the middle of the week and to decrease on weekends.

At the onset of time-step t, denote $z_i(t)$ be the number of new edges that connect to node v_i. First consider the directed GT model. Although the directed GT model defined by Eq. (2) does not completely determine G_t, it completely determines the in-degree statistics, which are enough for the estimation of A_k and η_i. So we abuse the notation a bit and write $P(G_t|G_{t-1}, m(t), n(t), \mathbf{A}, \eta)$ for the probability of the in-degree statistics in G_t given the conditional.

Now having defined the notation, the term $P(G_t|G_{t-1}, m(t), n(t), \mathbf{A}, \eta)$ is then a multinomial distribution probability. This comes from the observation that given $m(t)$, the quantities $z_1(t)$, $z_2(t)$, …, $z_N(t)$ follow a multinomial distribution with parameters $\pi_1(t)$, $\pi_2(t)$, …, $\pi_N(t)$, where $\pi_i(t)$, the probability that a newly added edge at time t connects to node v_i, is

$$\pi_i(t) = \frac{A_{k_i(t)}\eta_i}{\sum_{j=1}^{N} A_{k_j(t)}\eta_j}. \tag{S6}$$

Here we use two conventions: if node v_j does not exist in the network at time t, then $k_j(t) = -1$ and $A_{-1} = 0$. Note that both A_k and η_i are only identifiable up to a multiplicative constant, as can be seen from Eq. (S6). For the formal treatment in this section, we assume that $A_0 = 1$ and $\eta_1 = 1$. In practice, one can normalize A_k and η_i separately as one deems fit.

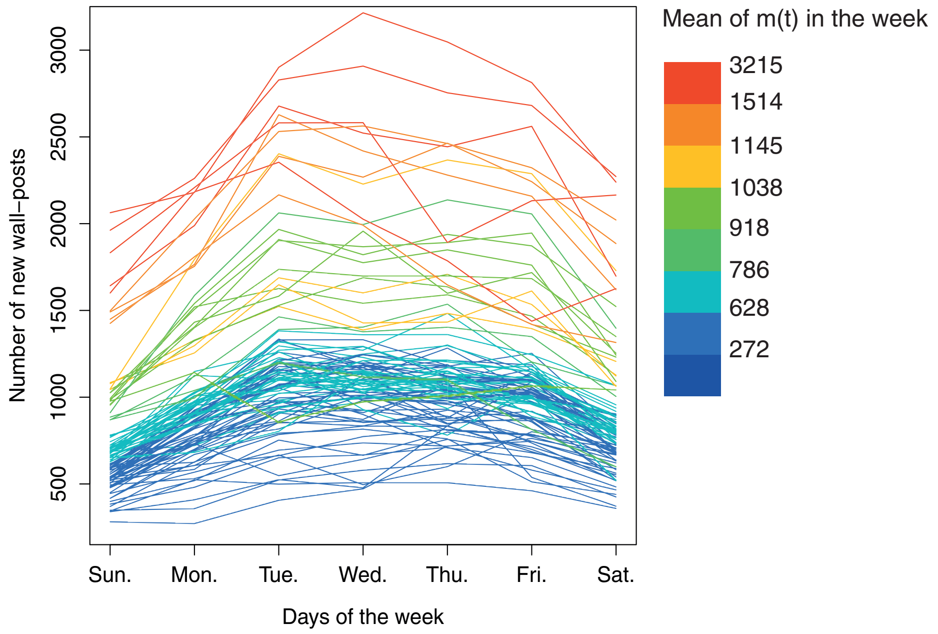

Supplementary Figure S10. The number of new wall-posts tends to increase toward middle of the week and decrease on weekends. Each curve presents the numbers of wall-posts of days in a week. The color of each curve is based on the mean of numbers of new wall-posts in that week.

We can write down the log-likelihood function:

$$\begin{aligned} l(\mathbf{A},\eta) &= \sum_{t=1}^{T} \log P\left(G_t | G_{t-1}, m(t), n(t), \mathbf{A}, \eta\right) \\ &= \sum_{t=1}^{T}\sum_{i=1}^{N} z_i(t) \log A_{k_i(t)} + \sum_{t=1}^{T}\sum_{i=1}^{N} z_i(t) \log \eta_i - \sum_{t=1}^{T}\sum_{i=1}^{N} z_i(t) \log \sum_{j=1}^{N} A_{k_j(t)}\eta_j + C, \end{aligned} \tag{S7}$$

with C being a constant that depends neither on $\mathbf{A}$ or η. We note about the concavity of the log-likelihood function. In general, $l(\mathbf{A},\eta)$ is not jointly concave in $\mathbf{A}$ and η. But using the same calculation as in Pham *et al.*[2], we can show that for any fixed value $\mathbf{A}^*$ of $\mathbf{A}$, $l(\mathbf{A}^*,\eta)$ is concave in η. Similarly, for any fixed value η^* of η, we can show the concavity of $l(\mathbf{A},\eta^*)$.

In the undirected GT model, the probability of an edge $e_{i,j}$ given by Eqs. (S3) and (S4) can be transformed as follows.

$$\begin{aligned} \pi_{i,j}(t) &= \frac{2^{I_{i\neq j}} A_{k_i(t)}\eta_i A_{k_j(t)}\eta_j}{\sum_{u=1}^{N}\sum_{q=1}^{N} A_{k_u(t)}\eta_u A_{k_q(t)}\eta_q} \\ &= 2^{I_{i\neq j}} \frac{A_{k_i(t)}\eta_i}{\sum_{u=1}^{N} A_{k_u(t)}\eta_u} \frac{A_{k_j(t)}\eta_j}{\sum_{q=1}^{N} A_{k_q(t)}\eta_q} \\ &= 2^{I_{i\neq j}} \pi_i(t)\pi_j(t), \end{aligned}$$

where I is the indicator function and $\pi_i(t)$ is defined in Eq. (S6).

For the case $i \neq j$, by viewing an undirected edge $e_{i,j}$ as two directed edges connect to v_i and v_j, we can see that the log-likelihood function in Eq. (S7) correctly captures the log-likelihood of $e_{i,j}$, up to an additive constant of $\log 2$. For the case $i = j$, since we interpret a loop as one directed edge, we miss an additional term of $\log \pi_i(t)$ in the log-likelihood function. Denote $l_{\text{undirect}}(\mathbf{A},\eta)$ as the log-likelihood of the undirected GT model, and $\zeta_i(t)$ as the number of self-loops of node v_i at time t. Recall that $l(\mathbf{A},\eta)$ is the log-likelihood of the converted directed network under the directed GT model. Then we have

$$l_{\text{undirect}}(\mathbf{A},\eta) = l(\mathbf{A},\eta) + \sum_t \sum_i \zeta_i(t) \log \pi_i(t), \tag{S8}$$

up to some constants. With little modifications, all of the algorithms described in the next section can be easily applied with $l(\mathbf{A},\eta)$ replaced by $l_{\text{undirect}}(\mathbf{A},\eta)$. From Eq. (S8), we can see that if there are no self-loops in the undirected network, then

$l(\mathbf{A}, \eta)$ is exactly the log-likelihood of the undirected network, and so the algorithms described in the next section can be applied without any modifications.

S2.4 Algorithms for maximizing the objective function

Here we describe an MM algorithm for maximizing the objective function $h(\mathbf{A}, \eta)$ in Eq. (3). The strategy here is the same as what can be found in Pham *et al.*[2] for the case of estimating A_k in isolation. First we find an MM algorithm for maximizing the log-likelihood function (Eq. (4)). The algorithm for maximizing $h(\mathbf{A}, \eta)$ is then based on this algorithm.

Solving the likelihood equation $\partial l/\partial \mathbf{A} = 0$ and $\partial l/\partial \eta = 0$, we obtain

$$A_k = \frac{\sum_{t=1}^{T} m_k(t)}{\sum_{t=1}^{T} \frac{m(t)}{\sum_{j=1}^{N} A_{k_j(t)}\eta_j} \sum_{i:k_i(t)=k} \eta_i} \quad \text{for } k \text{ from 1 to } K-1, \tag{S9}$$

$$\eta_i = \frac{\sum_{t=1}^{T} z_i(t)}{\sum_{t=1}^{T} \frac{m(t)}{\sum_{j=1}^{N} A_{k_j(t)}\eta_j} A_{k_i(t)}} \quad \text{for } i \text{ from 2 to } N. \tag{S10}$$

Since A_k and η_i appear in both sides of Eqs. (S9) and (S10), an explicit solution for $\mathbf{A}$ and η is difficult to obtain. In order to maximize Eq. (S7), we start from some initial value $\mathbf{A}^{(0)} = \left[1\ A_1^{(0)} \cdots A_{K-1}^{(0)}\right]$ and $\eta^{(0)} = \left[1\ \eta_2^{(0)} \cdots \eta_N^{(0)}\right]$ for $\mathbf{A}$ and η at step $q = 0$ and then iteratively update:

$$A_k^{(q+1)} \leftarrow \frac{\sum_{t=1}^{T} m_k(t)}{\sum_{t=1}^{T} \frac{m(t)}{\sum_{j=1}^{N} A_{k_j(t)}^{(q)}\eta_j^{(q)}} \sum_{i:k_i(t)=k} \eta_i^{(q)}} \quad \text{for } k \text{ from 1 to } K-1, \tag{S11}$$

and

$$\eta_i^{(q+1)} \leftarrow \frac{\sum_{t=1}^{T} z_i(t)}{\sum_{t=1}^{T} \frac{m(t)}{\sum_{j=1}^{N} A_{k_j(t)}^{(q+1)}\eta_j^{(q)}} A_{k_i(t)}^{(q+1)}} \quad \text{for } i \text{ from 2 to } N, \tag{S12}$$

until some convergence condition is met.

The running time of each iteration depends on the number of η_i and A_k we have to estimate. Recall that we often perform binning, in which we set $A_k = \omega_i$ for all k in the i-th bin. All algorithms described in this section remain valid with A_k's replaced by ω_i's. Assuming that the number of bins is B, the running time of each iteration is $O(TN + TB)$. The number of iterations needed depends on the convergence condition and the particular dataset.

In order to show that the algorithm consisting of Eqs. (S11) and (S12) can indeed increase the log-likelihood function $l(\mathbf{A}, \eta)$ at each iteration, we first find a minorize function $Q(\mathbf{A}, \eta)$ of l at the point $(\mathbf{A}^{(q)}, \eta^{(q)})$. Such a function Q, by definition[30], satisfies $Q \leq l$ at all points and $Q(\mathbf{A}^{(q)}, \eta^{(q)}) = l(\mathbf{A}^{(q)}, \eta^{(q)})$. Consider the function

$$Q(\mathbf{A}, \eta) = \sum_{t=1}^{T}\sum_{i=1}^{N} z_i(t)\log A_{k_i(t)} + \sum_{t=1}^{T}\sum_{i=1}^{N} z_i(t)\log \eta_i - \sum_{t=1}^{T}\sum_{i=1}^{N} z_i(t)\log \sum_{j=1}^{N} A_{k_j(t)}^{(q)}\eta_j^{(q)} - \sum_{t=1}^{T}\sum_{i=1}^{N} z_i(t)\frac{\sum_{j=1}^{N} A_{k_j(t)}\eta_j}{\sum_{j=1}^{N} A_{k_j(t)}^{(q)}\eta_j^{(q)}} + \sum_{t=1}^{T}\sum_{i=1}^{N} z_i(t). \tag{S13}$$

One can check that Q defined by Eq. (S13) is a minorize function of l. Next, it can be easily verified that Eq. (S11) is in fact the solution of $\partial Q/\partial \mathbf{A} = 0$, thus $Q(\mathbf{A}, \eta) \leq Q\left(\mathbf{A}^{(q+1)}, \eta\right)$. Finally, we have $l(\mathbf{A}^{(q)}, \eta^{(q)}) = Q(\mathbf{A}^{(q)}, \eta^{(q)}) \leq Q\left(\mathbf{A}^{(q+1)}, \eta^{(q)}\right) \leq l\left(\mathbf{A}^{(q+1)}, \eta^{(q)}\right)$, which shows that Eq. (S11) indeed increases the log-likelihood. In the same way we can show that Eq. (S12) further increases l from $l\left(\mathbf{A}^{(q+1)}, \eta^{(q)}\right)$.

Note that, the current algorithm (Eqs. (S11) and (S12)) first calculates $\mathbf{A}^{(q+1)}$, and then use $\mathbf{A}^{(q+1)}$ to calculate $\eta^{(q+1)}$. It is possible to calculate $\mathbf{A}^{(q+1)}$ and $\eta^{(q+1)}$ in parallel by modifying the current algorithm. Define

$$Q'(\mathbf{A},\eta) = \sum_{t=1}^{T}\sum_{i=1}^{N} z_i(t)\log A_{k_i(t)} + \sum_{t=1}^{T}\sum_{i=1}^{N} z_i(t)\log\eta_i - \sum_{t=1}^{T}\sum_{i=1}^{N} z_i(t)\log\sum_{j=1}^{N} A_{k_j(t)}^{(q)}\eta_j^{(q)} - \sum_{t=1}^{T}\sum_{i=1}^{N} z_i(t)\frac{\sum_{j=1}^{N}\frac{1}{2}\left(\frac{\eta_j^{(q)}}{A_{k_j(t)}^{(q)}}A_{k_j(t)}^2 + \frac{A_{k_j(t)}^{(q)}}{\eta_j^{(q)}}\eta_j^2\right)}{\sum_{j=1}^{N} A_{k_j(t)}^{(q)}\eta_j^{(q)}} + \sum_{t=1}^{T}\sum_{i=1}^{N} z_i(t),$$

then Q' is a minorize function of l at point $(\mathbf{A}^{(q)},\eta^{(q)})$. The key point here is that the two sets of equations $\partial Q'/\partial\mathbf{A} = 0$ and $\partial Q'/\partial\eta = 0$ are separable, so we can calculate $\mathbf{A}^{(q+1)}$ and $\eta^{(q+1)}$ in parallel by using Q' instead of Q. Nevertheless, we prefer the current algorithm in Eqs. (S11) and (S12), since cyclic updating of $\mathbf{A}$ and η is potentially faster than updating in parallel[30]. Furthermore, the update formulas in Eqs. (S11) and (S12) are also conceptually simpler.

Next we derive the update equation for η_i for the objective function in Eq. (3). The new formula for $\eta_i^{(q+1)}$ is similar to Eq. (S12), but with the discount from s added:

$$\eta_i^{(q+1)} \leftarrow \frac{\sum_{t=1}^{T} z_i(t) + s - 1}{\sum_{t=1}^{T} m(t)\frac{A_{k_i(t)}^{(q+1)}}{\sum_{j=1}^{N} A_{k_j(t)}^{(q+1)}\eta_j^{(q)}} + s},$$

except when $s < 1$, the estimated fitness of a node whose $\sum_{t=1}^{T} z_j(t) = 0$ is simply 0.

Finally, we derive the update for A_k when the regularization term in Eq. (5) is added. Although the same derivation can be found in Ref. 2, we also record it here for completeness. $A_k^{(q+1)}$ is the solution of a univariate equation which can be derived as follows. First we need to find a minorize function of Eq. (5). The strategy is the same as what can be found in Ref. 30. For a concave function $g(x)$, we have the following inequality that comes directly from the definition of a concave function.

$$g\left(\frac{1}{2}m + \frac{1}{4}n + \frac{1}{4}p\right) \geq \frac{1}{2}g(m) + \frac{1}{4}g(n) + \frac{1}{4}g(p).$$

By choosing $g(x) = -w_k x^2$ and

$$m = -4\log A_k + 2\log A_k^{(q)} + \log A_{k+1}^{(q)} + \log A_{k-1}^{(q)},$$
$$n = 4\log A_{k+1} + \log A_{k-1}^{(q)} - 2\log A_k^{(q)} - 3\log A_{k+1}^{(q)},$$
$$p = 4\log A_{k-1} - 2\log A_k^{(q)} + \log A_{k+1}^{(q)} - 3\log A_{k-1}^{(q)},$$

we have

$$\begin{aligned} & -r\sum_k w_k\left(\log A_{k+1} + \log A_{k-1} - 2\log A_k\right)^2 \\ & \geq -r\sum_k\left(\frac{w_k}{2}\left(-4\log A_k + 2\log A_k^{(q)} + \log A_{k+1}^{(q)} + \log A_{k-1}^{(q)}\right)^2 + \right. \\ & \frac{w_k}{4}\left(4\log A_{k+1} + \log A_{k-1}^{(q)} - 2\log A_k^{(q)} - 3\log A_{k+1}^{(q)}\right)^2 + \\ & \left.\frac{w_k}{4}\left(4\log A_{k-1} - 2\log A_k^{(q)} + \log A_{k+1}^{(q)} - 3\log A_{k-1}^{(q)}\right)^2\right). \end{aligned} \qquad \text{(S14)}$$

We can easily verify that the right hand side of Eq. (S14) is a minorize function of Eq. (5). Combining this function with the minorize function of the log-likelihood (see Eq. (S13)), we get a minorize function Q_A of $h(\mathbf{A},\eta)$ at iteration q. The main point of deriving Eq. (S14) is that by using Eq. (S14), at each iteration we can turn the $K+1$-variable maximization problem $\partial Q_A/\partial\mathbf{A} = 0$ into $K+1$ problems $\partial Q_A/\partial A_k = 0$ in which each is univariate and can be easily solved in parallel.

Supplementary References

1. Kong, J., Sarshar, N. & Roychowdhury, V. Experience versus talent shapes the structure of the web. *Proceedings of the National Academy of Sciences of the USA* **37**, 105 (2008).
2. Pham, T., Sheridan, P. & Shimodaira, H. PAFit: A statistical method for measuring preferential attachment in temporal complex networks. *PLOS ONE* e0137796 (2015).
3. Newman, M. Clustering and preferential attachment in growing networks. *Physical Review E* **64**, 025102 (2001).
4. Jeong, H., Néda, Z. & Barabási, A. Measuring preferential attachment in evolving networks. *Europhysics Letters* **61**, 567–572 (2003).
5. Massen, C. & Jonathan, P. Preferential attachment during the evolution of a potential energy landscape. *The Journal of Chemical Physics* **127**, 114306 (2007).
6. Sheridan, P., Yagahara, Y. & Shimodaira, H. Measuring preferential attachment in growing networks with missing-timelines using Markov chain Monte Carlo. *Physica A Statistical Mechanics and its Applications* **391**, 5031–5040 (2012).
7. Gómez, V., Kappen, H. J. & Kaltenbrunner, A. Modeling the structure and evolution of discussion cascades. In *Proceedings of the 22Nd ACM Conference on Hypertext and Hypermedia*, HT '11, 181–190 (ACM, New York, NY, USA, 2011).
8. Golosovsky, M. & Solomon, S. The transition towards immortality: Non-linear autocatalytic growth of citations to scientific papers. *Journal of Statistical Physics* **151**, 340–354 (2013).
9. Kunegis, J., Blattner, M. & Moser, C. Preferential attachment in online networks: Measurement and explanations. In *Proceedings of the 5th Annual ACM Web Science Conference*, WebSci '13, 205–214 (ACM, New York, NY, USA, 2013).
10. Zhang, Q.-M., Xu, X.-K., Zhu, Y.-X. & Zhou, T. Measuring multiple evolution mechanisms of complex networks. *Scientific Reports* (2015).
11. Middendorf, M., Ziv, E. & Wiggins, C. H. Inferring network mechanisms: The drosophila melanogaster protein interaction network. *Proceedings of the National Academy of Sciences of the United States of America* **102**, 3192–3197 (2005).
12. Blasio, B. F. d., Seierstad, T. G. & Aalen, O. O. Frailty effects in networks: comparison and identification of individual heterogeneity versus preferential attachment in evolving networks. *Journal of the Royal Statistical Society: Series C (Applied Statistics)* **60**, 239–259 (2011).
13. Dorogovtsev, S. N. & Mendes, J. F. F. Evolution of networks with aging of sites. *Phys. Rev. E* **62**, 1842–1845 (2000).
14. Amaral, L. A. N., Scala, A., Barthélémy, M. & Stanley, H. E. Classes of small-world networks. *Proceedings of the National Academy of Sciences* **97**, 11149–11152 (2000).
15. Dorogovtsev, S. N. & Mendes, J. F. F. *Evolution of Networks: From Biological Nets to the Internet and WWW (Physics)* (Oxford University Press, Inc., New York, NY, USA, 2003).
16. Csardi, G., Strandburg, K., Zalanyi, L., Tobochnik, J. & Erdi, P. Modeling innovation by a kinetic description of the patent citation system. *Physica A* **374**, 783 – 793 (2007).
17. Medo, M. c. v., Cimini, G. & Gualdi, S. Temporal effects in the growth of networks. *Phys. Rev. Lett.* **107**, 238701 (2011).
18. Wang, M., Yu, G. & Yu, D. Measuring the preferential attachment mechanism in citation networks. *Physica A: Statistical Mechanics and its Applications* **387**, 4692 – 4698 (2008).
19. Wang, D., Song, C. & Barabási, A.-L. Quantifying long-term scientific impact. *Science* **342**, 127–132 (2013).
20. Shen, H.-W., Wang, D., Song, C. & Barabási, A. Modeling and predicting popularity dynamics via reinforced poisson processes. In *Proceedings of The Twenty-Eighth AAAI Conference on Artificial Intelligence* (2014).
21. Chatterjee, S., Diaconis, P. & Sly, A. Random graphs with a given degree sequence. *Ann. Appl. Probab.* **21**, 1400–1435 (2011).
22. Holland, P. W. & Leinhardt, S. An exponential family of probability distributions for directed graphs. *Journal of the American Statistical Association* **76**, pp. 33–50 (1981).
23. Bradley, R. A. & Terry, M. E. Rank analysis of incomplete block designs: I. the method of paired comparisons. *Biometrika* **39**, pp. 324–345 (1952).
24. Simons, G. & Yao, Y.-C. Asymptotics when the number of parameters tends to infinity in the Bradley-Terry model for paired comparisons. *Ann. Statist.* **27**, 1041–1060 (1999).
25. Hunter, D. R. MM algorithms for generalized Bradley-Terry models. *Ann. Statist.* **32**, 384–406 (2004).

26. Yan, T. & Xu, J. A central limit theorem in the β-model for undirected random graphs with a diverging number of vertices. *Biometrika* **100**, 519–524 (2013).

27. Rinaldo, A., Petrović, S. & Fienberg, S. E. Maximum lilkelihood estimation in the β-model. *Ann. Statist.* **41**, 1085–1110 (2013).

28. Móri, T. F. The maximum degree of the Barabási-Albert random tree. *Comb. Probab. Comput.* **14**, 339–348 (2005).

29. Móri, T. F. On a 2-parameter class of scale free random graphs. *Acta Mathematica Hungarica* **114**, 37–48 (2007).

30. Hunter, D. & Lange, K. A tutorial on MM algorithms. *The American Statistician* **58**, 30–37 (2004).
